# Diagnostic imaging modalities in Dyspnoea and suspected acute heart failure: a systematic review with Meta-analysis^☆^

**DOI:** 10.1016/j.ijcha.2026.102015

**Published:** 2026-09-21

**Authors:** Anne Sophie Overgaard Olesen, Kristina Miger, Ida Arentz Taraldsen, Jasmin Lukoschewitz, Emilie Melgaard, Emma Malm, Sandra Tonning, Lucas Westergaard, Simone Haastrup, Mohammed El-Sheikh, Olav Wendelboe Nielsen, Jens Jakob Thune, Pardeep S. Jhund, Alasdair D. Henderson, Janus Christian Jakobsen, Johannes Grand

**Affiliations:** a Department of Cardiology, Copenhagen University Hospital – Bispebjerg and Frederiksberg, Copenhagen, Denmark; b Department of Clinical Medicine, University of Copenhagen, Copenhagen, Denmark; c British Heart Foundation Cardiovascular Research Centre, School of Cardiovascular and Metabolic Health, University of Glasgow, Glasgow, United Kingdom; d Department of Cardiology, Copenhagen University Hospital – Amager and Hvidovre, Copenhagen, Denmark; e Copenhagen Trial Unit, Centre for Clinical Intervention Research, Copenhagen University Hospital - Rigshospitalet, Copenhagen, Denmark; f Department of Regional Health Research, Faculty of Health Sciences, University of Southern Denmark, Odense, Denmark

**Keywords:** Acute heart failure, Pulmonary congestion, Lung ultrasound, Chest radiography, Computed tomography, Diagnostic accuracy

## Abstract

**Background:**

Early identification of pulmonary congestion in patients with dyspnoea is critical for managing acute heart failure. However, the optimal imaging modality remains uncertain. We compared chest radiography, lung ultrasound, computed tomography (CT), and remote dielectric sensing (ReDS) for detecting acute heart failure.

**Methods:**

We conducted a preregistered systematic review and meta-analysis of prospective observational studies in adults with dyspnoea. Medline, Embase, LILACS, and Web of Science were searched on April 9, 2025. Two reviewers independently screened studies, extracted data, and assessed bias. Sensitivity and specificity were pooled using bivariate random-effects models. Comparative performance was assessed by Bayesian network meta-analysis with weakly informative priors.

**Results:**

We included 95 studies (15,132 patients). Lung ultrasound was evaluated in 78 studies (11,790 patients), chest radiography in 30 (5966), CT in two (368), and ReDS in six (525). Some studies evaluated multiple modalities. Pooled sensitivity and specificity were 0.87 (95%CI: 0.82–0.90) and 0.89 (0.85–0.91) for lung ultrasound, 0.65 (0.58–0.72) and 0.91 (0.87–0.94) for chest radiography, 0.73 (0.53–0.87) and 0.73 (0.58–0.84) for ReDS. CT was not included in bivariate modelling because data were sparse; Bayesian network estimates were sensitivity 0.76 (95%CrI 0.62–0.86) and specificity 0.94 (0.88–0.97). CT and lung ultrasound each had >95% posterior probability of higher sensitivity than chest radiography and higher specificity than both chest radiography and ReDS.

**Conclusion:**

Lung ultrasound may serve as a first-line bedside modality. A normal chest radiograph cannot reliably exclude acute heart failure. CT showed high specificity based on limited evidence. The role of ReDS remains uncertain.

## Highlights

• Acute dyspnoea requires rapid identification of acute heart failure.
• We compared chest radiography, lung ultrasound, computed tomography, and ReDS.
• Lung ultrasound had the highest sensitivity in suspected acute heart failure.
• Computed tomography showed the highest specificity, but evidence was limited.
• Lung ultrasound may be preferred for first-line bedside assessment.

## Graphical abstract

**Figure:**
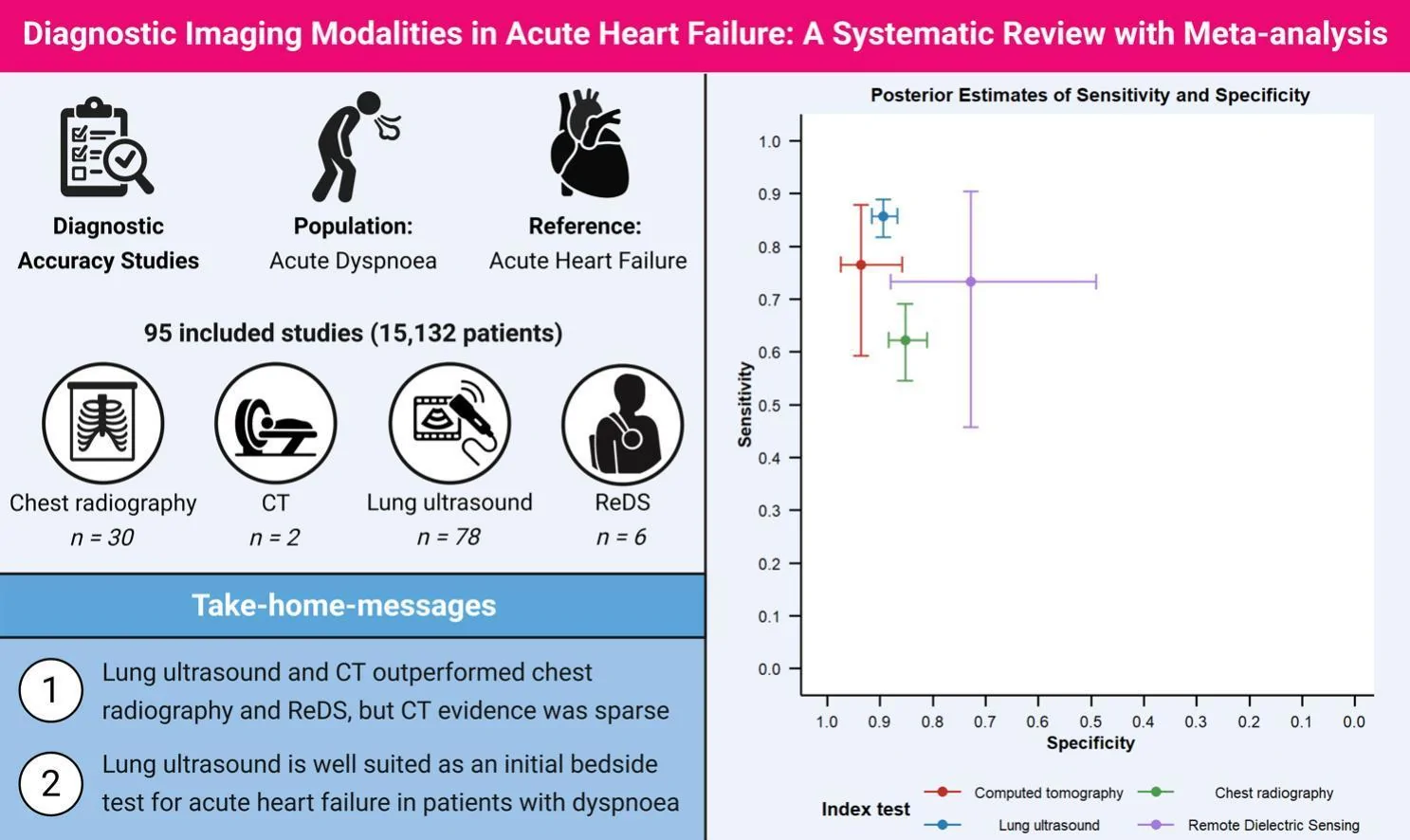
Diagnostic performance of chest radiography, lung ultrasound, computed tomography, and remote dielectric sensing for detecting acute heart failure in patients with acute dyspnoea.

## 1 Introduction

Dyspnoea is a leading cause of emergency admissions, with acute heart failure accounting for up to half of cases [1], [2]. Pulmonary congestion, which is present in up to three quarters of patients, is a major cause of dyspnoea in acute heart failure [3]. Early recognition of pulmonary congestion is therefore essential, as timely guideline-directed treatment of acute heart failure can prevent deterioration and improve survival [4], [5]. Detecting pulmonary congestion is challenging as dyspnoea is non-specific and may arise from pulmonary conditions such as chronic obstructive pulmonary disease or pneumonia [6]. Echocardiography is recommended to confirm acute heart failure but primarily evaluates cardiac structure, function, and haemodynamics, rather than pulmonary congestion itself and is often unavailable during the initial assessment. Clinicians in acute care therefore rely on other readily available methods to assess patients presenting with acute dyspnoea to guide management. Clinical examination and chest radiography remain routine, but their limited accuracy leads to misdiagnosis in one in five patients presenting with acute dyspnoea [7].

Lung ultrasound provides a rapid, radiation-free bedside assessment of pulmonary congestion [5], [8]. However, the B-lines seen in pulmonary congestion are not specific to heart failure and also occur in pneumonia and interstitial lung disease [8]. Although lung ultrasound is recommended in guidelines [8], it is not consistently integrated into routine diagnostic pathways [9]. Computed tomography (CT) provides excellent anatomical detail and may distinguish between cardiac and pulmonary causes of dyspnoea [10]. Moreover, low-dose techniques have increased its use in emergency care [11], [12]. Remote Dielectric Sensing (ReDS) is a novel electromagnetic-based technology that estimates lung fluid content within minutes [13]. No prior study has performed a meta-analysis evaluating and comparing traditional and emerging methods for detecting pulmonary congestion in acute heart failure.

### 1.1 Aim

This systematic review with meta-analysis aimed to evaluate and compare the diagnostic performance of chest radiography, lung ultrasound, CT, and ReDS for detecting pulmonary congestion and supporting the diagnosis of acute heart failure in adults presenting with acute dyspnoea. We estimated pooled sensitivities and specificities for each method and evaluated the methodological quality.

## 2 Methods

We followed the Preferred Reporting Items for Systematic Reviews and Meta-Analysis (PRISMA) [14] and the Cochrane Handbook for Systematic Reviews of Diagnostic Test Accuracy [15]. The study was registered with PROSPERO (CRD420250651927) and the protocol was published before the literature search [16]. The protocol addressed both pulmonary congestion and pleural effusion. Pleural effusion will be reported separately because it involves clinically distinct populations beyond acute heart failure.

### 2.1 Search strategy and selection criteria

We searched Medical Literature Analysis and Retrieval System Online (MEDLINE Ovid), Excerpta Medica database (Embase Ovid), Latin American and Caribbean Health Sciences Literature (VHL Regional Portal), Science Citation Index Expanded (Web of Science), Conference Proceedings Citation Index-Science (Web of Science). We had no restrictions on language or publication year. The search took place on April 9, 2025, and the search strategies are provided in Supplemental Methods 1. Reference lists of included studies and relevant reviews were screened.

Eligible studies were prospective diagnostic accuracy studies enrolling adults aged 18 years or older with dyspnoea or suspected acute heart failure in emergency, in-hospital, intensive care, or prehospital settings. Studies were included if they evaluated one of the four modalities against an investigator-defined reference standard for pulmonary congestion or acute heart failure and reported data allowing construction of a 2 × 2 table or calculation of sensitivity, specificity, or diagnostic accuracy. When multiple publications used the same cohort, the most complete dataset was included, and head-to-head comparisons were prioritised.

### 2.2 Data collection and risk of bias

Two reviewers independently screened records, extracted data, and assessed risk of bias and applicability, with disagreements resolved by consensus or a third reviewer [16]. Extracted variables included study setting, population, index test protocol, reference standard, and diagnostic accuracy data. Risk of bias and applicability were assessed using QUADAS-2 [17], covering patient selection, index test, reference standard, and flow and timing. Briefly, these domains assess whether patient selection was representative, whether the index test was conducted and interpreted appropriately, whether the reference standard accurately and independently identified the target condition, and whether all participants received comparable reference testing within an appropriate interval. Each domain was rated as low, high, or unclear risk of bias, and studies were classified as high or unclear overall risk if any domain was rated high or unclear. Publication bias was assessed using Deeks' funnel plots when at least 10 studies were available.

### 2.3 Outcome and data analysis

The primary outcomes were sensitivity and specificity for each index test. For all studies, 2 × 2 contingency data were reconstructed and cross-checked against reported estimates. Authors were contacted when essential information was missing, and studies with unresolved missing data were included only in analyses where this was possible. All statistical analyses were conducted in R version 4.4.2.

The primary analysis used a bivariate hierarchical summary receiver operating characteristic (HSROC) model to generate pooled sensitivity, specificity, likelihood ratios, and diagnostic odds ratios with 95% confidence intervals (CIs). Analyses were performed using the MetaDTA web application [18], [19]. Secondary analyses used univariate random-effects models to estimate sensitivity, specificity, positive and negative predictive values, and diagnostic accuracy, defined as the proportion of correctly classified cases. Studies reporting overall diagnostic accuracy but insufficient data to reconstruct 2 × 2 tables were included in the accuracy analyses only.

We applied hierarchical random-effects models following Cochrane guidance for diagnostic accuracy reviews [15], reflecting expected variation across populations, settings, and reference standards. Fixed-effect estimates are presented for comparison. For CT, both random-effects and fixed-effect estimates are reported because the evidence base was particularly sparse. Bivariate modelling was precluded. CT was nevertheless included in the Bayesian network model used for cross-modality comparison, which jointly estimated all modalities and accounted for dependence between modalities evaluated within the same study. The Bayesian and univariate CT estimates are therefore not expected to be identical.

### 2.4 Bayesian network meta-analysis

To compare index tests, we performed a Bayesian network meta-analysis, as the prespecified likelihood-ratio-based pairwise comparisons proved infeasible due to sparse or missing head-to-head data [16]. We fitted a bivariate binomial model using the *brms* package [20] to estimate the sensitivity and specificity from each study with a fixed effect for each index test, and a study-level random effect. Unlike the conventional bivariate models, which were fitted separately for each modality, the Bayesian model estimated all modalities jointly and accounted for dependence between modalities evaluated within the same study. Estimates from the two approaches were therefore not expected to be identical. We used weakly informative priors, conducted prior predictive checks, convergence diagnostics and performed multiple sensitivity analyses with alternative priors and in selected subgroups. Full details are in Supplemental Methods 2. Posterior means and 95%-credible intervals (CrI) were reported for each modality. Pairwise posterior differences were examined to determine whether the 95%CrI included 0.

### 2.5 Assessment of heterogeneity

Statistical heterogeneity was assessed using multiple approaches. For univariate analyses, between-study heterogeneity was quantified using the I^2^ statistic. Given the limitations of I^2^ in diagnostic accuracy meta-analyses [15], heterogeneity was also evaluated by visual inspection of forest plots, HSROC space, and summary confidence and prediction regions.

Given the limited number of head-to-head comparisons and network sparsity, formal assessment of transitivity was not feasible. As transitivity is an underlying assumption of network meta-analysis, we explored potential sources of clinical and methodological heterogeneity through subgroup and sensitivity analyses by reference standard and setting. Network-derived estimates were further compared with results from head-to-head analyses where available.

### 2.6 Subgroup analyses by reference standards and settings

Because reference standards varied across studies, prespecified subgroup analyses were performed by reference standard. Reference standards were grouped into the following categories: diagnosis of acute heart failure (*n* = 42), presence of pulmonary oedema (*n* = 37), natriuretic peptide-based criteria (*n* = 3), investigator determined cardiogenic dyspnoea (*n* = 3), and invasive cardiac catheterisation measures (*n* = 6) of extravascular lung water index, pulmonary artery wedge pressure, and left ventricular end-diastolic pressure. To further explore clinical heterogeneity, additional subgroup analyses were performed according to clinical setting.

## 3 Results

The search identified 156,101 studies, of which 118 prospective studies met the inclusion criteria (Supplemental Fig. S1). Of these, we included the 95 studies that evaluated pulmonary congestion or acute heart failure, totalling 15,132 patients. Among these studies, 78 evaluated lung ultrasound, 30 chest radiography, two examined CT, and six evaluated ReDS. Of these studies, 20 assessed both lung ultrasound and chest radiography, and one assessed both CT and chest radiography (Supplemental Fig. S2, Supplemental References). Four studies, three evaluating lung ultrasound and one evaluating ReDS, reported overall accuracy but insufficient data to reconstruct 2 × 2 tables and therefore contributed only to the accuracy analyses.

Study sample sizes ranged from 19 to 1005 participants and mean/median ages ranged from 35 to 86 years. Inclusion and exclusion criteria, index test protocols, reference standards, and baseline variables are provided in Supplemental Tables S1-S5.

### 3.1 Lung ultrasound

In the bivariate model, lung ultrasound showed a pooled sensitivity of 0.87 (95%CI: 0.82–0.90) and specificity of 0.89 (95%CI: 0.85–0.91), corresponding to a diagnostic odds ratio of 49.4 (95%CI: 32.4–75.4) (Table 1; Fig. 1). Univariate random-effects analyses produced the same pooled sensitivity and specificity, with I^2^ = 88% for both (Supplemental Fig. S3). Additional univariate measures, including accuracy, predictive values and fixed effect models, are presented in Supplemental Figs. S3-S4. In 72% of studies, risk of bias was rated as high or unclear in patient selection, 62% in the reference standard domain, and 41% in flow and timing.

**Table 1:** Summary of bivariate meta-analysis results for index tests.

Index test	Sensitivity (95%-CI)	Specificity (95%-CI)	Diagnostic odds ratio (95%-CI)	LR+ (95%-CI)	LR- (95%-CI)
**Lung ultrasound**	0.87 (0.82–0.90)	0.89 (0.85–0.91)	49.4 (32.4–75.4)	7.5 (5.9–9.6)	0.15 (0.11–0.20)
**Chest radiography**	0.65 (0.58–0.72)	0.91 (0.87–0.94)	18.2 (12.0–27.4)	7.0 (5.0–9.9)	0.39 (0.32–0.47)
**ReDS**	0.73 (0.53–0.87)	0.73 (0.58–0.84)	7.3 (2.6–20.4)	2.7 (1.6–4.6)	0.37 (0.20–0.70)

CT was evaluated in only two studies and therefore not included in the model.

ReDS: Remote Dielectric Sensing, LR+: Positive likelihood ratio, LR-: Negative likelihood ratio.

**Fig. 1:**
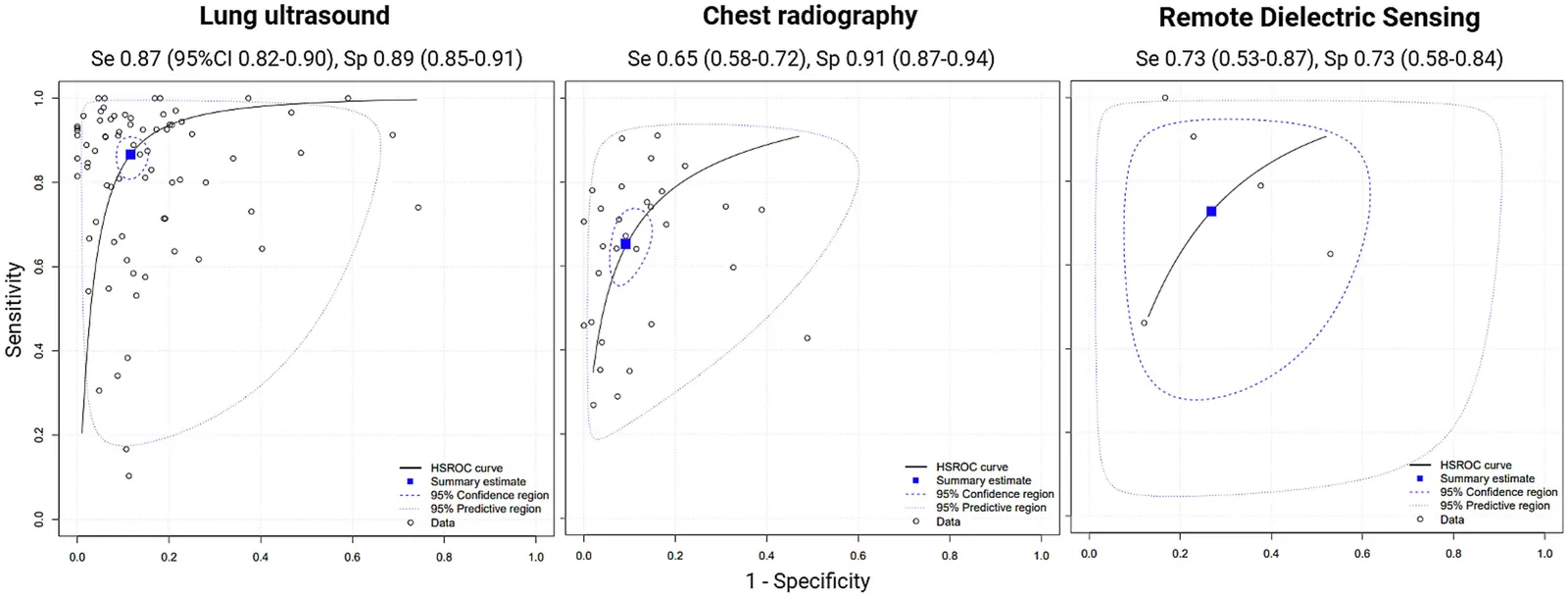
Summary ROC curves from bivariate models **Legend:***Hierarchical summary receiver operating characteristic curves generated from bivariate meta-analytic models. Each panel shows the summary estimate (■), the 95% credible region around the summary point (dashed line), and the 95% predictive region (dotted line) indicating expected variation in future studies. Open circles represent data from individual studies.*

### 3.2 Chest radiography

For chest radiography, the bivariate analysis yielded a pooled sensitivity of 0.65 (95%CI: 0.58–0.72) and a specificity of 0.91 (95%CI: 0.87–0.94), corresponding to a diagnostic odds ratio of 18.2 (95%CI: 12.0–27.4) (Table 1; Fig. 1). In the univariate random-effects models, the pooled estimates were identical to those from the bivariate analysis, with I^2^ values of 93% for sensitivity and 84% for specificity (Supplemental Fig. S5). Additional univariate estimates are provided in Supplemental Figs. S5-S6. In 73% of studies, risk of bias was high or unclear in the domain of patient selection, 83% in the reference standard domain, and 50% in flow and timing.

### 3.3 CT

Only two studies evaluated CT, precluding bivariate modelling. In univariate random-effects meta-analyses, pooled sensitivity and specificity were 0.99 (95%CI: 0.15–1.00; I^2^ = 0%) and 0.96 (95%CI: 0.92–0.98; I^2^ = 0%) (Supplemental Fig. S7). Fixed-effect estimates were 0.90 (95%CI: 0.85–0.94) for sensitivity and 0.96 (95%CI: 0.92–0.98) for specificity (Supplemental Fig. S8). Both studies were overall at high or unclear risk of bias.

### 3.4 Remote dielectric sensing

In the bivariate model, ReDS had a pooled sensitivity of 0.73 (95%CI: 0.53–0.87) and specificity of 0.73 (95%CI: 0.58–0.84), corresponding to a diagnostic odds ratio of 7.3 (95%CI: 2.6–20.4) (Table 1; Fig. 1). Univariate analyses yielded the same pooled estimates, with I^2^ values of 79% for sensitivity and 82% for specificity (Supplemental Fig. S9). Additional univariate estimates are provided in Supplemental Figs. S9-S10. The risk of bias was high or unclear for all studies in at least one domain.

### 3.5 Bayesian network meta-analysis

Posterior mean estimates from the Bayesian model for lung ultrasound were a sensitivity of 0.86 (95%CrI: 0.82–0.88) and specificity 0.89 (95%CrI: 0.87–0.91), chest radiography sensitivity 0.62 (95%CrI: 0.56–0.68) and specificity 0.85 (95%CrI: 0.82–0.88), CT sensitivity 0.76 (95%CrI: 0.62–0.86) and specificity 0.94 (95%CrI: 0.88–0.97), and ReDS had sensitivity 0.73 (95%CrI: 0.50–0.87) and specificity 0.73 (95%CrI: 0.53–0.86) (Fig. 2, Fig. 3; Supplemental Fig. S11).

**Fig. 2:**
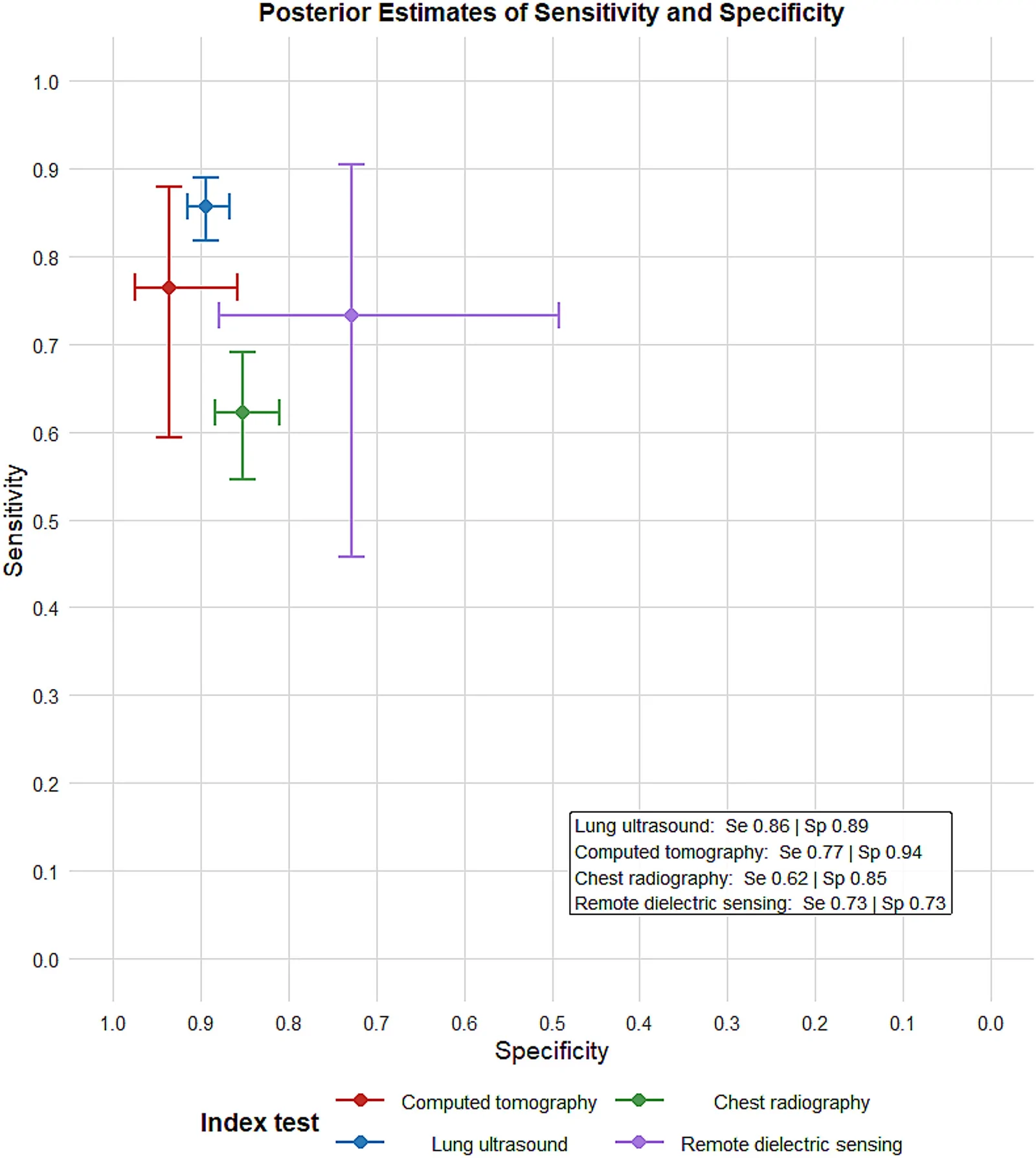
Bayesian network meta-analysis of sensitivity and specificity across index tests **Legend:***Posterior mean sensitivity and specificity with 95% credible intervals from the Bayesian bivariate network meta-analysis.*

**Fig. 3:**
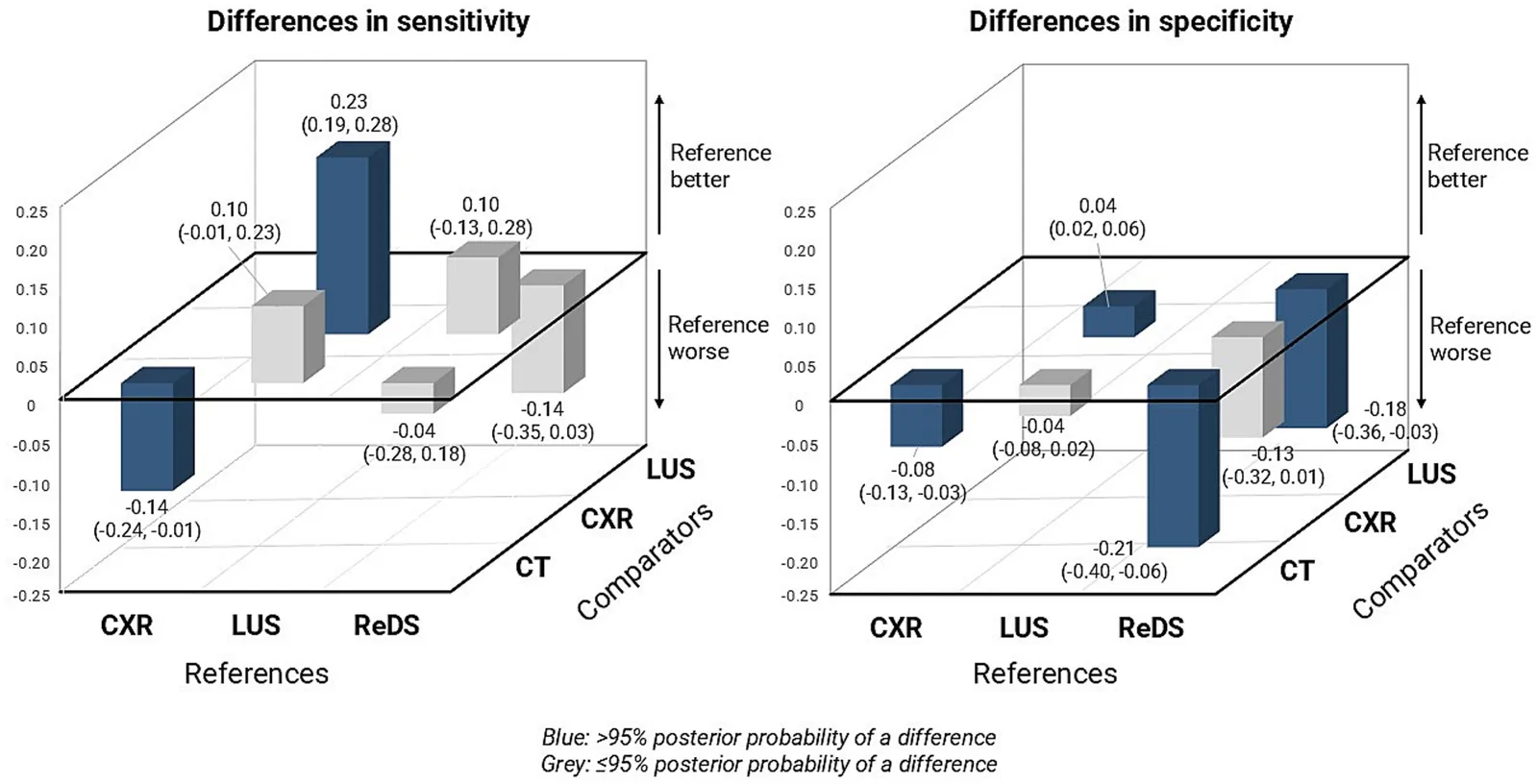
Pairwise differences in posterior mean sensitivity and specificity **Legend:***Positive bars reflect differences favouring the reference test, and negative bars reflect differences favouring the comparator. Blue bars indicate pairwise comparisons with a >* *95% posterior probability of a difference. Differences are presented with 95% credible intervals.* CXR: chest radiography, LUS: lung ultrasound, CT: computed tomography, ReDS: remote dielectric sensing.

Pairwise comparisons showed >95% posterior probability that CT had higher posterior mean sensitivity than chest radiography (mean difference 0.14, 95%CrI: 0.01–0.24) and higher specificity than both chest radiography (0.08, 95%CrI: 0.03–0.13) and ReDS (0.21, 95%CrI: 0.06–0.40) (Fig. 3). Lung ultrasound showed higher sensitivity than chest radiography (0.23, 95%CrI: 0.19–0.28) and higher specificity than chest radiography (0.04, 95%CrI: 0.02–0.06) and ReDS (0.18, 95%CrI: 0.03–0.36).

For comparisons where the 95% credible intervals included 0, we report the posterior probability that one modality outperformed another. Lung ultrasound had a high probability of greater sensitivity than both CT (94%) and ReDS (89%). CT showed a high probability of greater specificity than lung ultrasound (90%) and a modest probability of greater sensitivity than ReDS (59%). ReDS was more sensitive than chest radiography in 79% of draws, while chest radiography was more specific than ReDS in 93%.

Model diagnostics confirmed good convergence and R^ values of 1.00–1.01 (Supplemental Fig. S12) and robustness across alternative prior specifications (Supplemental Table S6, Supplemental Fig. S13–14).

### 3.6 Heterogeneity

Substantial between-study heterogeneity was observed, with high I^2^ values for sensitivity and specificity for lung ultrasound, chest radiography and ReDS (Supplemental Figs. S3-S10). Visual inspection of forest plots showed broadly overlapping confidence intervals, with only a small number of outlying studies for lung ultrasound. For these, we found no clear explanation when reviewing reference standards, settings, or study quality. In bivariate analyses, heterogeneity was reflected by wide confidence and prediction regions in HSROC space (Fig. 1).

### 3.7 Subgroup analysis by trialist-defined reference standard and setting

For lung ultrasound, univariate pooled sensitivity ranged from 0.72 to 0.89 and varied significantly across reference standards (*P* = 0.027), driven primarily by lower sensitivities in studies using invasive hemodynamic criteria. Specificity was more consistent (0.89–0.91, *P* = 0.166). For chest radiography, variation in sensitivity and specificity across reference standards did not reach statistical significance (*P* ≥ 0.092) (Supplemental Figs. S15-S16). CT and ReDS were not analysed due to the limited number of available studies.

Nine studies defined pulmonary congestion exclusively by CT findings, which could introduce dependency as CT also served as an index test (Supplemental Table 2S). Repeating the network analysis without these studies produced estimates identical to the primary analysis, except for a minimal increase in chest radiography sensitivity (0.62 to 0.64). All pairwise comparisons remained unchanged.

Across settings (emergency department, intensive care unit (ICU), wards, prehospital), pooled sensitivity and specificity for lung ultrasound and chest radiography were similar (Supplemental Figs. S17-S18).

### 3.8 Subgroup analysis of head-to-head studies

Among 20 studies that evaluated chest radiography and lung ultrasound within the same patient populations, pooled sensitivity and specificity were 0.90 and 0.89 for lung ultrasound and 0.67 and 0.90 for chest radiography (Supplemental Fig. S19).

### 3.9 Risk of bias

Only four studies were rated as low risk of bias in all domains (Supplemental Fig. S20). Patient selection showed the fewest low-risk assessments (26%), while flow and timing performed best (59% low risk) (Fig. 4). Chest radiography had the lowest index-test quality (32% low risk). Applicability concerns mainly related to patient selection (48% judged appropriate). Funnel plots suggested small-study bias for lung ultrasound and chest radiography but were not performed for CT or ReDS because fewer than 10 studies were available (Supplemental Figs. S21-S22).

**Fig. 4:**
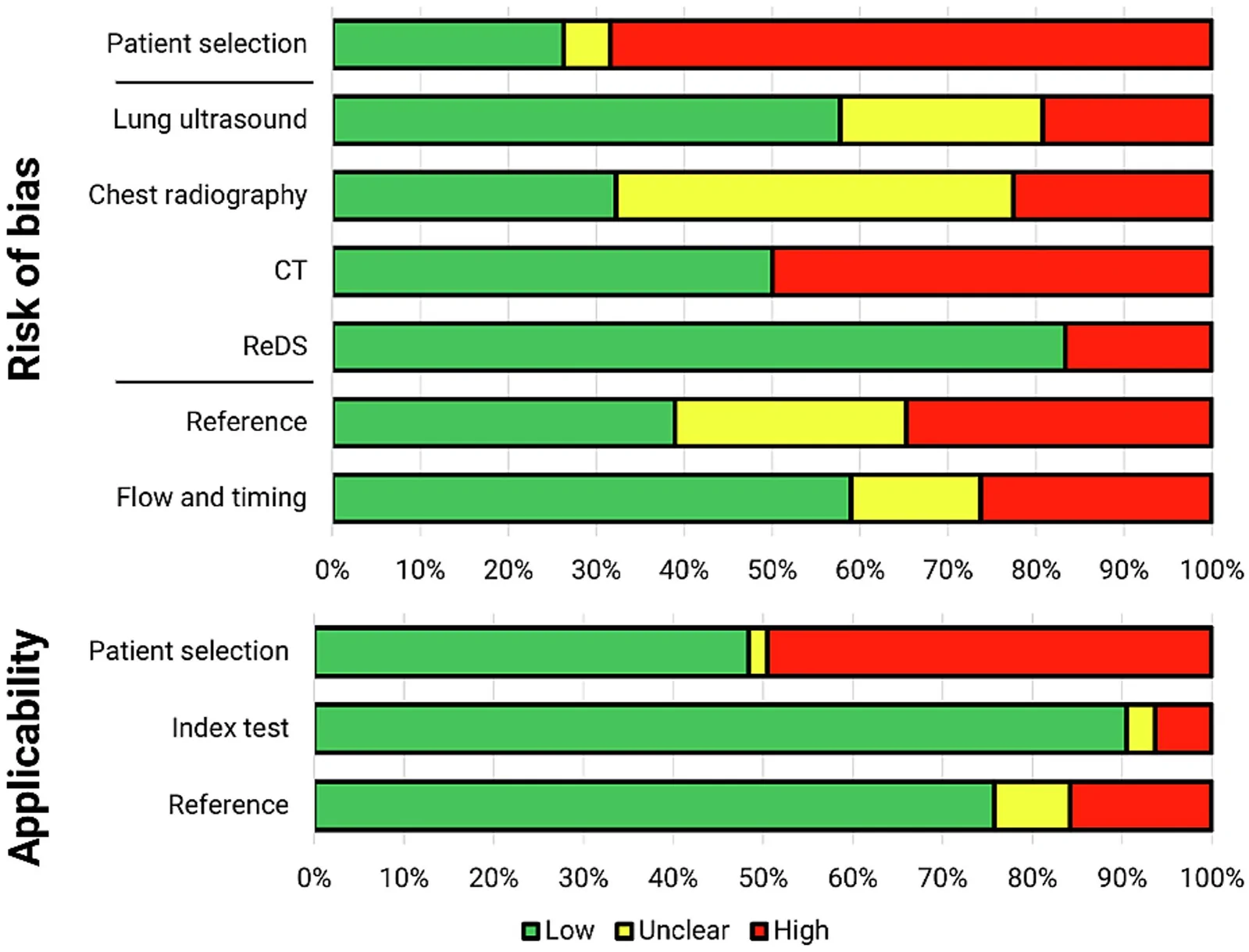
Risk of bias and applicability concerns **Legend:***Assessment based on QUADAS-2 domains.*

## 4 Discussion

In this systematic review with network meta-analysis of 95 studies including over 15,000 patients, lung ultrasound showed the most consistently favourable diagnostic profile for detecting pulmonary congestion in patients with suspected acute heart failure, combining the highest sensitivity with greater accuracy than chest radiography and higher specificity than ReDS. CT had the highest estimated specificity, but this finding was based on only two studies. Overall, the results support lung ultrasound as the leading first-line bedside modality in this setting, although the high or unclear risk of bias in most studies limits the certainty of the evidence.

To our knowledge, this is the first network meta-analysis comparing imaging modalities for detecting pulmonary congestion in suspected acute heart failure. Previous reviews mainly evaluated lung ultrasound alone [21], [22] or compared lung ultrasound with chest radiography in six and eight head-to-head studies, consistently favouring lung ultrasound [23], [24]. As prespecified, we included prospective diagnostic accuracy studies because randomised diagnostic strategy trials are scarce [25], [26], [27], and establishing test accuracy is necessary before evaluating effects on treatment decisions or outcomes.

The high sensitivity of lung ultrasound, combined with its bedside availability, absence of radiation, and potential for repeated assessment, supports its use as a first-line test in patients with acute dyspnoea and suspected acute heart failure. It may also complement natriuretic peptides, which are widely used but can be influenced by comorbidities and may not always provide clear rule-in or rule-out information in the acute setting [5]. Nevertheless, B-lines are not specific to heart failure and may occur in interstitial lung disease or pneumonia, while early or mild congestion may yield false-negative findings [8]. Performance may also depend on operator training, scanning protocol, and clinical integration [8]. Chest radiography remains widely available and inexpensive, but its limited sensitivity precludes use as a stand-alone rule-out test. Instead, it may serve as an adjunct when lung ultrasound is unavailable. However, the frequent incorporation of radiographic findings into the reference standard contributed to a high risk of bias in several studies evaluating chest radiography. Among the less established modalities, CT had the highest specificity and may support the diagnosis of acute heart failure when bedside imaging is inconclusive or alternative pathology is suspected. However, the evidence was limited to only two studies, and one of the two studies (Inui et al.) had potential incorporation bias because CT findings contributed to the composite reference diagnosis. Sensitivity estimates also varied across modelling approaches, likely reflecting both the limited amount of data and the resulting uncertainty in model-based estimates. The CT estimates should therefore be interpreted cautiously. ReDS was included to reflect the growing interest in rapid, non-invasive wearable technologies for assessing pulmonary fluid [28]. In our meta-analysis, ReDS demonstrated moderate diagnostic accuracy, likely because its dielectric signal reflects total thoracic fluid and may be influenced by emphysema or fibrosis [29]. Further studies are therefore needed to define the clinical roles of both CT and ReDS in undifferentiated dyspnoea.

Several limitations should be acknowledged. First, studies varied in index-test protocols, patient populations, and reference standards, which included clinical diagnoses of acute heart failure, pulmonary congestion, natriuretic peptide criteria, and invasive haemodynamic measurements. These reference standards capture related but distinct aspects of disease. In particular, pulmonary congestion is a pathophysiological manifestation that commonly occurs in acute heart failure but may also occur in other conditions, while acute heart failure is a clinical syndrome diagnosed using symptoms, signs, biomarkers, cardiac imaging, and the clinical course. This variation may have influenced diagnostic performance, although subgroup analyses showed broadly similar findings across most reference-standard definitions, and head-to-head analyses showed consistent findings for lung ultrasound and chest radiography. However, lung ultrasound had lower sensitivity in studies using invasive haemodynamic criteria. This may reflect that haemodynamic congestion, indicated by elevated cardiac filling pressures, can occur before pulmonary interstitial fluid accumulation becomes detectable by lung ultrasound. Future studies of acute heart failure require agreed, standardised diagnostic criteria and well-designed prospective trials with blinded adjudication of the final diagnosis. Second, patients with acute dyspnoea often have alternative or concurrent causes, and the imaging modalities provide complementary rather than interchangeable information. Chest radiography and lung ultrasound may identify pneumonia or pneumothorax, whereas CT provides a broader assessment of pulmonary pathology. Although this additional diagnostic information was beyond the scope of our analysis, it influences how imaging is used alongside clinical assessment, biomarkers, and echocardiography in practice. However, estimating the diagnostic contribution of each modality remains an important step before imaging-based diagnostic strategies can be evaluated in clinical trials. Third, the certainty of the evidence was limited by small-study effects for lung ultrasound and chest radiography and by the high or unclear risk of bias in most studies. This included non-representative patient selection, such as the exclusion of older adults and patients with chronic lung disease, which may have inflated performance estimates for modalities sensitive to interstitial abnormalities, including lung ultrasound and ReDS. Finally, the time elapsed since the systematic search represents a limitation. A targeted search for eligible CT and ReDS diagnostic-accuracy studies published since April 9, 2025 did not identify any new eligible studies likely to materially affect the results.

This review also has several strengths. It represents the largest synthesis of diagnostic accuracy studies to date and includes patients from emergency departments, wards, ICUs, and prehospital settings. The analyses followed a preregistered protocol, used rigorous quality assessment, and combined both conventional bivariate models with Bayesian network meta-analysis, providing robust and consistent estimates across modalities.

## 5 Conclusion

Lung ultrasound may be considered the preferred initial modality for detecting pulmonary congestion in patients with acute dyspnoea and suspected acute heart failure. Because of its limited sensitivity for pulmonary congestion, a normal chest radiograph cannot reliably exclude acute heart failure. CT showed favourable specificity but was supported by few studies, and current evidence does not support routine use of ReDS.

## Funding statements

The study did not receive funding, but some of the authors' salaries during the work was supported by the following not-for-profit funds: Novo Nordisk Foundation (NNF22OC0079241) (Drs Olesen and Haastrup), and Danish Cardiovascular Academy, which is funded by the Novo Nordisk Foundation and The Danish Heart Foundation (Drs Taraldsen, Miger and Grand). The funders had no role in study design, data collection/analysis, manuscript preparation, or the decision to publish.

## Declaration of generative AI and AI-assisted technologies

During the preparation of this work, the authors used ChatGPT-5.2 for language revisions of the methods and discussion sections only. After using this tool, the authors reviewed and edited the content as needed and take full responsibility for the content of the manuscript.

## CRediT authorship contribution statement

**Anne Sophie Overgaard Olesen:** Writing – review & editing, Writing – original draft, Visualization, Validation, Supervision, Software, Resources, Project administration, Methodology, Investigation, Formal analysis, Data curation, Conceptualization. **Kristina Miger:** Writing – review & editing, Visualization, Validation, Methodology, Investigation, Data curation, Conceptualization. **Ida Arentz Taraldsen:** Writing – review & editing, Validation, Methodology, Investigation, Data curation. **Jasmin Lukoschewitz:** Writing – review & editing, Validation, Methodology, Investigation, Data curation. **Emilie Melgaard:** Writing – review & editing, Validation, Methodology, Investigation, Data curation. **Emma Malm:** Writing – review & editing, Validation, Methodology, Investigation, Data curation. **Sandra Tonning:** Writing – review & editing, Validation, Methodology, Investigation, Data curation. **Lucas Westergaard:** Writing – review & editing, Validation, Methodology, Investigation, Data curation. **Simone Haastrup:** Writing – review & editing, Validation, Methodology, Investigation, Data curation. **Mohammed El-Sheikh:** Writing – review & editing, Validation, Methodology, Investigation, Data curation. **Olav Wendelboe Nielsen:** Writing – review & editing, Visualization, Supervision, Methodology, Conceptualization. **Jens Jakob Thune:** Writing – review & editing, Visualization, Supervision, Methodology, Conceptualization. **Pardeep S. Jhund:** Writing – review & editing, Visualization, Supervision, Methodology, Conceptualization. **Alasdair D. Henderson:** Writing – review & editing, Visualization, Supervision, Software, Methodology, Formal analysis, Conceptualization. **Janus Christian Jakobsen:** Writing – review & editing, Visualization, Supervision, Resources, Methodology, Investigation, Conceptualization. **Johannes Grand:** Writing – review & editing, Visualization, Supervision, Resources, Methodology, Investigation, Conceptualization.

## Declaration of competing interest

The authors declare the following financial interests/personal relationships which may be considered as potential competing interests: Anne Sophie Overgaard Olesen reports financial support was provided by Novo Nordisk Foundation. Olav Wendelboe Nielsen reports a relationship with Novo Nordisk that includes: employment. If there are other authors, they declare that they have no known competing financial interests or personal relationships that could have appeared to influence the work reported in this paper.

## Data Availability

All data used in this systematic review were extracted from previously published studies, and no individual patient data were collected. The authors are willing to share the extracted dataset and analysis code upon reasonable request.

## Appendix A Supplementary data

Supplementary material 1 Supplemental appendix containing supplemental figures, tables, methods, and references.
