# Supplementary material for: Diagnostic imaging modalities in Dyspnoea and suspected acute heart failure: a systematic review with Meta-analysis
Source: Int J Cardiol Heart Vasc. 2026 Sep 21;67:102015. doi: 10.1016/j.ijcha.2026.102015 (PMC13628669; doi:10.1016/j.ijcha.2026.102015)
Supplement: Supplementary file 1 [file mmc1.docx]

**Supplemental Appendix**

[**Supplemental Figures**	3](#_Toc220585611)

[Supplemental Figure 1. Study selection process	4](#_Toc220585612)

[Supplemental Figure 2. Network plot	5](#_Toc220585613)

[Supplemental Figure 3a. Forest plots of pooled univariate estimates for lung ultrasound	6](#_Toc220585614)

[Supplemental Figure 3b. Forest plots of pooled univariate estimates for lung ultrasound	7](#_Toc220585615)

[Supplemental Figure 3c. Forest plots of pooled univariate estimates for lung ultrasound	8](#_Toc220585616)

[Supplemental Figure 4. Forest plots of pooled univariate estimates for lung ultrasound using fixed effect	9](#_Toc220585617)

[Supplemental Figure 5a. Forest plots of pooled univariate estimates for chest radiography	10](#_Toc220585618)

[Supplemental Figure 5b. Forest plots of pooled univariate estimates for chest radiography	11](#_Toc220585619)

[Supplemental Figure 5c. Forest plots of pooled univariate estimates for chest radiography	12](#_Toc220585620)

[Supplemental Figure 6. Forest plots of pooled univariate estimates for chest radiography using fixed effect model	13](#_Toc220585621)

[Supplemental Figure 7. Forest plots of pooled univariate estimates for CT	14](#_Toc220585622)

[Supplemental Figure 8. Forest plots of pooled univariate estimates for CT using fixed effect model	15](#_Toc220585623)

[Supplemental Figure 9. Forest plots of pooled univariate estimates for ReDS	16](#_Toc220585624)

[Supplemental Figure 10. Forest plots of pooled univariate estimates for ReDS using fixed effect model	17](#_Toc220585625)

[Supplemental Figure 11. Posterior estimates and 95% credible regions across index tests from Bayesian network meta-analysis	18](#_Toc220585626)

[Supplemental Figure 12. Trace plots for Bayesian model convergence	19](#_Toc220585627)

[Supplemental Figure 13. Prior predictive checks	20](#_Toc220585628)

[Supplemental Figure 14. Prior robustness assessment using power-scaling	21](#_Toc220585629)

[Supplemental Figure 15. Stratified univariate meta-analysis of lung ultrasound by trialist-chosen reference standards	22](#_Toc220585630)

[Supplemental Figure 16. Stratified univariate meta-analysis of chest radiography by trialist-chosen reference standards	22](#_Toc220585631)

[Supplemental Figure 17. Stratified univariate meta-analysis of lung ultrasound by trial setting	23](#_Toc220585632)

[Supplemental Figure 18. Stratified univariate meta-analysis of chest radiography by trial setting	23](#_Toc220585633)

[Supplemental Figure 19. Univariate sensitivity and specificity in head-to-head studies for chest radiography and lung ultrasound	24](#_Toc220585634)

[Supplemental Figure 20. Risk of bias	25](#_Toc220585635)

[Supplemental Figure 21. Funnel plots and Deeks’ test for publication bias in studies evaluating chest radiography	29](#_Toc220585636)

[Supplemental Figure 22. Funnel plots and Deeks’ test for publication bias in studies evaluating lung ultrasound	30](#_Toc220585637)

[**Supplemental Tables**	31](#_Toc220585638)

[Supplemental Table 1a. Study characterizations for ReDS studies	32](#_Toc220585639)

[Supplemental Table 1b. Study characterizations for CT studies	32](#_Toc220585640)

[Supplemental Table 1c. Study characterizations for lung ultrasound studies	33](#_Toc220585641)

[Supplemental Table 1d. Study characterizations for chest radiography studies	39](#_Toc220585642)

[Supplemental Table 2. Study information on index test and reference	41](#_Toc220585643)

[Supplemental Table 3. Comorbidities and Symptoms	51](#_Toc220585644)

[Supplemental Table 4. Admission variables	56](#_Toc220585645)

[Supplemental Table 5. Admission Causes	60](#_Toc220585646)

[Supplemental Table 6. Sensitivity analyses of prior specifications for the Bayesian analysis	64](#_Toc220585647)

[**Supplemental Methods**	65](#_Toc220585648)

[Supplemental Methods 1. Search strategies	66](#_Toc220585649)

[Supplemental Methods 2. Bayesian model specification	69](#_Toc220585650)

[**Supplemental References**	72](#_Toc220585651)

# **Supplemental Figures**

## **Supplemental Figure 1.** Study selection process

**Identification**

References removed **(n = 2741)**

Duplicates identified manually (n = 160)

Duplicates identified by Covidence (n = 2581)

Studies from databases/registers **(n = 156101)**

Studies excluded **(n = 152349)**

Studies not retrieved **(n = 4)**

Full-text studies assessed for eligibility

**(n = 1007)**

Studies sought for retrieval **(n = 1011)**

Studies screened by title and abstract

**(n = 153360)**

**Screening**

Studies excluded **(n = 889)**

Not original research article (e.g., review, congress abstract) (n = 214)

Wrong setting (n = 20)

Wrong outcomes (n = 293)

Wrong index test (n = 51)

Wrong study design (n = 237)

Wrong patient population (n = 49)

Insufficient diagnostic accuracy data (n = 23)

Duplicated patient populations (n = 2)

**Included**

Studies included in review **(n = 118)**

## **Supplemental Figure 2.** Network plot

*Each node represents an index test, with node size proportional to the total number of studies including that test. Lines indicate direct comparisons between tests, with line thickness proportional to the number of studies comparing the connected tests.*

*CXR: chest radiography, LUS: lung ultrasound, CT: computed tomography, ReDS: remote dielectric sensing*

## **Supplemental Figure 3a.** Forest plots of pooled univariate estimates for lung ultrasound

## **Supplemental Figure 3b.** Forest plots of pooled univariate estimates for lung ultrasound

## **Supplemental Figure 3c.** Forest plots of pooled univariate estimates for lung ultrasound

## **Supplemental Figure 4.** Forest plots of pooled univariate estimates for lung ultrasound using fixed effect

## **Supplemental Figure 5a.** Forest plots of pooled univariate estimates for chest radiography

## **Supplemental Figure 5b.** Forest plots of pooled univariate estimates for chest radiography

## **Supplemental Figure 5c.** Forest plots of pooled univariate estimates for chest radiography

## **Supplemental Figure 6.** Forest plots of pooled univariate estimates for chest radiography using fixed effect model

## **Supplemental Figure 7.** Forest plots of pooled univariate estimates for CT

## **Supplemental Figure 8.** Forest plots of pooled univariate estimates for CT using fixed effect model

## **Supplemental Figure 9.** Forest plots of pooled univariate estimates for ReDS

## **Supplemental Figure 10.** Forest plots of pooled univariate estimates for ReDS using fixed effect model

## **Supplemental Figure 11.** Posterior estimates and 95% credible regions across index tests from Bayesian network meta-analysis

*Posterior sensitivity and specificity with 95% credible regions in ROC space from the Bayesian bivariate network meta-analysis of CT, chest radiography, lung ultrasound, and ReDS*

## **Supplemental Figure 12.** Trace plots for Bayesian model convergence

*Trace plots for the four Markov chains from the Bayesian bivariate random-effects network meta-analysis, showing posterior sampling for the logit-transformed sensitivity and specificity across index tests.*

## **Supplemental Figure 13.** Prior predictive checks

*Prior predictive distributions of true positives (A) and true negatives (B) from 100 hypothetical tests, generated from the priors before observing data.*

## **Supplemental Figure 14.** Prior robustness assessment using power-scaling

*Each panel shows the posterior density for a model parameter under weaker (blue), original (black), and stronger (red) priors. Minimal differences between the curves indicate that posterior estimates are robust to reasonable changes in prior strength, confirming that the priors are weakly informative relative to the data.*

## **Supplemental Figure 15.** Stratified univariate meta-analysis of lung ultrasound by trialist-chosen reference standards

*Pooled sensitivity and specificity of chest radiography by grouped reference standards (Acute heart failure = 34, Pulmonary oedema = 32, Brain natriuretic based = 2, Cardiogenic dyspnoea = 3, Invasive hemodynamics = 4 studies).*

## **Supplemental Figure 16.** Stratified univariate meta-analysis of chest radiography by trialist-chosen reference standards

*Pooled sensitivity and specificity of chest radiography by grouped reference standards (Acute heart failure = 16, Pulmonary oedema = 12, Cardiogenic dyspnoea = 1, Invasive hemodynamics = 1 studies).*

## **Supplemental Figure 17.** Stratified univariate meta-analysis of lung ultrasound by trial setting

*Pooled sensitivity and specificity of lung ultrasound by grouped trial settings (Emergency department = 44, Ward = 10, Intensive care unit = 17, Prehospital = 4 studies).*

## **Supplemental Figure 18.** Stratified univariate meta-analysis of chest radiography by trial setting

*Pooled sensitivity and specificity of chest radiography by grouped trial settings (Emergency department = 21, Ward = 3, Intensive care unit = 6 studies).*

**Supplemental Figure 19.** Univariate sensitivity and specificity in head-to-head studies for chest radiography and lung ultrasound

## **Supplemental Figure 20.** Risk of bias

| **Study** | **Risk of Bias** | | | | | | | **Applicability Concerns** | | |
| --- | --- | --- | --- | --- | --- | --- | --- | --- | --- | --- |
|  | **Patient selection** | **Lung ultrasound** | **Chest radiography** | **CT** | **ReDS** | **Reference standard** | **Flow and timing** | **Patient selection** | **Index test** | **Reference standard** |
| Aggarwal 2016 |  |  |  |  |  |  |  |  |  |  |
| Ali 2022 |  |  |  |  |  |  |  |  |  |  |
| Anderson 2013 |  |  |  |  |  |  |  |  |  |  |
| Arthur 2022 |  |  |  |  |  |  |  |  |  |  |
| Azab 2022 |  |  |  |  |  |  |  |  |  |  |
| Baid 2022 |  |  |  |  |  |  |  |  |  |  |
| Baker 2016 |  |  |  |  |  |  |  |  |  |  |
| Baldi 2012 |  |  |  |  |  |  |  |  |  |  |
| Balen 2020 |  |  |  |  |  |  |  |  |  |  |
| Bataille 2014 |  |  |  |  |  |  |  |  |  |  |
| Bekgoz 2019 |  |  |  |  |  |  |  |  |  |  |
| Bitar 2015 |  |  |  |  |  |  |  |  |  |  |
| Bouzidi 2024 |  |  |  |  |  |  |  |  |  |  |
| Buessler 2019 |  |  |  |  |  |  |  |  |  |  |
| Buhumaid 2019 |  |  |  |  |  |  |  |  |  |  |
| Carlino 2018 |  |  |  |  |  |  |  |  |  |  |
| Chaitra 2021 |  |  |  |  |  |  |  |  |  |  |
| Chiem 2015 |  |  |  |  |  |  |  |  |  |  |
| Cibinel 2011 |  |  |  |  |  |  |  |  |  |  |
| Coiro 2015 |  |  |  |  |  |  |  |  |  |  |
| De Carvalho 2020 |  |  |  |  |  |  |  |  |  |  |
| Dehbozorgi 2019 |  |  |  |  |  |  |  |  |  |  |
| Dexheimer Neto 2015 |  |  |  |  |  |  |  |  |  |  |
| Elatroush 2023 |  |  |  |  |  |  |  |  |  |  |
| Elmahalawy 2017 |  |  |  |  |  |  |  |  |  |  |
| Elnaem 2017 |  |  |  |  |  |  |  |  |  |  |
| Ely 2001 |  |  |  |  |  |  |  |  |  |  |
| Farahmand 2020 |  |  |  |  |  |  |  |  |  |  |
| Feuerstein 2022 |  |  |  |  |  |  |  |  |  |  |
| Gallard 2015 |  |  |  |  |  |  |  |  |  |  |
| Gargani 2008 |  |  |  |  |  |  |  |  |  |  |
| Ghauri 2021 |  |  |  |  |  |  |  |  |  |  |
| Gillespie 1997 |  |  |  |  |  |  |  |  |  |  |
| Glöckner 2020 |  |  |  |  |  |  |  |  |  |  |
| Heldeweg 2022 |  |  |  |  |  |  |  |  |  |  |
| Hubert 2019 |  |  |  |  |  |  |  |  |  |  |
| Imamura 2022 |  |  |  |  |  |  |  |  |  |  |
| Inui G 2024 |  |  |  |  |  |  |  |  |  |  |
| Izumida 2023 |  |  |  |  |  |  |  |  |  |  |
| Izumida 2022 |  |  |  |  |  |  |  |  |  |  |
| Johannessen 2023 |  |  |  |  |  |  |  |  |  |  |
| Joseph 2024 |  |  |  |  |  |  |  |  |  |  |
| Kennedy 2011 |  |  |  |  |  |  |  |  |  |  |
| Knudsen 2004 |  |  |  |  |  |  |  |  |  |  |
| Koh 2018 |  |  |  |  |  |  |  |  |  |  |
| Lajili 2024 |  |  |  |  |  |  |  |  |  |  |
| Laursen 2016 |  |  |  |  |  |  |  |  |  |  |
| Lichtenstein 2008 |  |  |  |  |  |  |  |  |  |  |
| Lichtenstein 2009 |  |  |  |  |  |  |  |  |  |  |
| Liteplo 2009 |  |  |  |  |  |  |  |  |  |  |
| Liu 2016 |  |  |  |  |  |  |  |  |  |  |
| Martinez-Rumayer 2009 |  |  |  |  |  |  |  |  |  |  |
| Mayr 2022 |  |  |  |  |  |  |  |  |  |  |
| Miger K 2019 |  |  |  |  |  |  |  |  |  |  |
| Miger K 2024 |  |  |  |  |  |  |  |  |  |  |
| Modi 2024 |  |  |  |  |  |  |  |  |  |  |
| Msolli 2021 |  |  |  |  |  |  |  |  |  |  |
| Mueller-Lenke N 2006 |  |  |  |  |  |  |  |  |  |  |
| Mumoli N 2016 |  |  |  |  |  |  |  |  |  |  |
| Nagaharu K 2022 |  |  |  |  |  |  |  |  |  |  |
| Nakao 2021 |  |  |  |  |  |  |  |  |  |  |
| Neeraj 2023 |  |  |  |  |  |  |  |  |  |  |
| Ocel S 2024 |  |  |  |  |  |  |  |  |  |  |
| Öhman 2019 |  |  |  |  |  |  |  |  |  |  |
| Olesen 2022 |  |  |  |  |  |  |  |  |  |  |
| Panda R 2023 |  |  |  |  |  |  |  |  |  |  |
| Patel CJ 2018 |  |  |  |  |  |  |  |  |  |  |
| Perrone 2017 |  |  |  |  |  |  |  |  |  |  |
| Pivetta 2015 |  |  |  |  |  |  |  |  |  |  |
| Prosen 2011 |  |  |  |  |  |  |  |  |  |  |
| Rafique 2022 |  |  |  |  |  |  |  |  |  |  |
| Russell F 2017 |  |  |  |  |  |  |  |  |  |  |
| Russell F 2023 |  |  |  |  |  |  |  |  |  |  |
| Saboo 2022 |  |  |  |  |  |  |  |  |  |  |
| Sartini 2017 |  |  |  |  |  |  |  |  |  |  |
| Schoeneck 2021 |  |  |  |  |  |  |  |  |  |  |
| Secko 2022 |  |  |  |  |  |  |  |  |  |  |
| Seibel A 2020 |  |  |  |  |  |  |  |  |  |  |
| Sekiguchi H 2015 |  |  |  |  |  |  |  |  |  |  |
| Sen 2017 |  |  |  |  |  |  |  |  |  |  |
| Sforza A 2017 |  |  |  |  |  |  |  |  |  |  |
| Sharma 2025 |  |  |  |  |  |  |  |  |  |  |
| Siddiqi M 2025 |  |  |  |  |  |  |  |  |  |  |
| Studler U 2008 |  |  |  |  |  |  |  |  |  |  |
| Tækker M 2022 |  |  |  |  |  |  |  |  |  |  |
| Tsverava 2010 |  |  |  |  |  |  |  |  |  |  |
| Uriel 2018 |  |  |  |  |  |  |  |  |  |  |
| Vauthier 2021 |  |  |  |  |  |  |  |  |  |  |
| Vitturi N 2011 |  |  |  |  |  |  |  |  |  |  |
| Volpicelli 2014 |  |  |  |  |  |  |  |  |  |  |
| Wooten 2018 |  |  |  |  |  |  |  |  |  |  |
| Yang F 2017 |  |  |  |  |  |  |  |  |  |  |
| Zhong L 2012 |  |  |  |  |  |  |  |  |  |  |
| Zhou S 2014 |  |  |  |  |  |  |  |  |  |  |
| Zhu L 2024 |  |  |  |  |  |  |  |  |  |  |

## **Supplemental Figure 21.** Funnel plots and Deeks’ test for publication bias in studies evaluating chest radiography

## **Supplemental Figure 22.** Funnel plots and Deeks’ test for publication bias in studies evaluating lung ultrasound

# **Supplemental Tables**

| Supplemental Table 1a. Study characterisations for ReDS studies | | | | | | | | | |
| --- | --- | --- | --- | --- | --- | --- | --- | --- | --- |
| Study | **Country** | **Setting** | **Enrollment period** | **Index test(s)** | **Inclusion criteria** | **Exclusion criteria** | **No. enrolled** | **Age, Mean (SD)/Median [IQR**] | **Male, %** |
| Imamura T 2022 | Japan | Ward | 2021 | ReDS | Patients with chronic HF hospitalised for worsening symptoms, underwent RHC and ReDS after stabilisation | Mechanical circulatory support, unstable clinical condition, or too large body habitus for ReDS | 30 | 79 [73-84] | 43 |
| Izumida T 2022 | Japan | Ward | 2021-2022 | ReDS | Hospitalised HF patients, clinically stable condition, able to wear ReDS | Inappropriate physique for ReDS vest, dependent on hemodialysis | 117 | 77 [71-84] | 53 |
| Izumida T 2023 | Japan | Ward | NA | ReDS | Consecutive patients hospitalised for HF, diagnosed by Framingham criteria | Interstitial pneumonia, lung cancer, COPD, prior chest radiation | 19 | 77 [58-82] | 68 |
| Olesen AS 2022 | Denmark | ED | 2018-2019 | ReDS | Acute dyspnoea, age ≥50 years, one abnormal objective parameter supporting respiratory imbalance | Intensive care or ventilation, ACS, no consent, study examination after 12h from admission | 97 | 73 [71-75] | 61 |
| Rafique Z 2022 | USA | ED | 2017-2020 | ReDS | Age ≥ 21 years, chief complaint of dyspnoea, able to provide informed consent | Chest trauma, discomfort or improper fit of vest, anatomical abnormalities, implanted devices, pregnancy, prisoner, BMI <22 or >36, height <5′1″ or >6′4″ | 123 | 57 (12) | 59 |
| Uriel N 2018 | USA | Ward | 2016-2017 | ReDS | Consecutive patients with HF diagnosed by Framingham criteria, undergoing RHC (excluding mechanical support) | Height <155 or >190 cm, BMI <22 or >36, thoracic deformity, lesions aggravated by vest, intrathoracic mass or congenital abnormality, pacemaker, or vulnerable population (pregnant, lactating, unable to consent). | 139 | 55 (13) | 72 |
| Supplemental Table 1b. Study characterisations for CT studies | | | | | | | | | |
| Inui G 2024 | Japan | ED | 2015-2017 | CT | Diagnosed in the ED with community-acquired pneumonia (CAP) or pulmonary oedema, hospitalizsd within 24 hours, underwent CT and BNP measurement. | Patients with cardiogenic shock, pneumonia, non-cardiogenic pulmonary oedema, or acute coronary heart disease. Cases mimicking CAP if there was no improvement with antibiotic treatment. | 140 | NA | 49 |
| Miger K 2024 | Denmark | ED | 2017-2019 | CT, CXR | Age ≥50, acute dyspnoea, ≥1 abnormal parameter: RR >20/min, SpO₂ <95% (<92% if COPD), O₂ need, signs of HF (bilateral rales, leg oedema), rhonchi, prolonged expiration | ICU/inotropes/ventilation need, ACS, dementia/language barrier, not Danish citizen, life expectancy <3 mo, refusal, CT already indicated for PE/aortic disease, CT/echo not possible within 12h | 228 | 74 [NA] | 57 |
| Supplemental Table 1c. Study characterisations for lung ultrasound studies | | | | | | | | | |
| Aggarwal M 2016 | India | ED, ICU | 2014 | LUS, CXR | Age 20–80 y, acute onset dyspnoea and fatigue, structural heart disease, or MI with LV dysfunction | Renal dysfunction, dialysis need, stage D heart failure, chest wall injuries, serious comorbidities (e.g., liver disease, ARDS, pneumonia), inability to consent | 42 | 54 (NA) | 62 |
| Ali M 2022 | Egypt | ED | 2020-2021 | LUS, CXR | Age >18 y; chief complaint of dyspnoea and/or chest pain | Unstable tachyarrhythmia or bradyarrhythmia causing shock; known diagnosis from referring hospital | 100 | 53 (14) | 59 |
| Anderson KL 2013 | USA | ED | NA | LUS | Presented to the ED with acute dyspnoea, suspected CHF exacerbation by treating physician, presence of 1 of 5 investigators. | <18 y, unable to consent | 101 | 62 [53-91] | 52 |
| Arthur M 2023 | India | Ward | 2016-2017 | LUS | ≥18 y, inpatient with new-onset respiratory or cardiac symptoms/signs, chest X-ray done | Pregnant, subcutaneous emphysema over the chest | 321 | 49 (NA) | 55 |
| Azab NY 2022 | Egypt | Ward | NA | LUS, CXR | Adults with confirmed acute respiratory failure (type I or II) | Children, patient refusal, incomplete data | 50 | 53 (16) | 56 |
| Baid H 2022 | India | ED | 2020-2021 | LUS | Acute onset shortness of breath; age >18 y | Referred patients with known diagnosis, dyspnoea due to trauma, pregnant | 237 | 53 [NA] | 60 |
| Baker K 2016 | Australia | ED | 2011-2012 | LUS, CXR | ≥60 years presenting to ED with any complaint that included shortness of breath | LUS interfered with resuscitation, trauma-related symptoms, chest X-ray not done within 1h (2h without fluid management), missing/indeterminate data. | 204 | 76 [69-83] | 54 |
| Baldi G 2013 | Italy | ICU | NA | LUS | ICU patients undergoing chest CT as part of clinical management | Age <18 y, LUS not feasible (e.g. trauma, burns), no informed consent | 20 | 66 [NA] | 65 |
| Balen F 2021 | France | ED | 2015-2019 | LUS | Age ≥ 65, dyspnoea, RR ≥ 25/min or SpO₂ < 90%, possible AHF | Life-threatening emergencies, SBP < 90 mmHg, STEMI RV, inability/refusal to consent | 116 | 84 (9) | 38 |
| Bataille B 2014 | France | ICU | 2012-2013 | LUS | Age ≥ 18 y and acute respiratory failure justifying ICU admission: RR ≥25/min, PaO₂ <60 mmHg, SpO₂ <90% on room air, PaCO₂ <45 mmHg with pH <7.35 | Patients with multiple final diagnoses; patients with acute exacerbation of chronic pulmonary disease were excluded from analysis | 136 | 68 (15) | 58 |
| Bekgoz 2019 | Turkey | ED | 2012-2013 | LUS | Adults >18 y presenting to ED with acute dyspnoea who consented | Cardiac arrest, trauma, pneumonectomy, interstitial lung disease, tracheal stenosis, fat embolism | 383 | 66 (16) | 52 |
| Bitar Z 2015 | Kuwait | ED | NA | LUS | Age >18 y, acute dyspnoea, clinical suspicion of pulmonary oedema/LV failure; NT-proBNP ordered in ED | Non-KOC members (due to lack of follow-up) and patients unable to consent | 61 | 67 (NA) | 49 |
| Bouzidi H 2025 | Tunisia | ED | 2022-2023 | LUS, CXR | Adult patients (≥18 y), acute dyspnoea, suspected cardiac origin (signs like tachycardia, rales, jugular distension, hepatojugular reflux, hepatomegaly, oedema) | Dyspnoea following trauma, BMI >40, history of pneumothorax, lobectomy, lung cancer, severe interstitial lung disease | 160 | 69 (13) | 56 |
| Buessler A 2019 | France | ED | 2015-2017 | LUS | Adults >50 y, admitted with acute dyspnoea, the treating physician had diagnostic uncertainty based on initial clinical evaluation. | Traumatic dyspnoea, systolic blood pressure <70 mmHg. | 117 | 80 (12) | 44 |
| Buhumaid RE 2019 | USA | ED | NA | LUS, CXR | Age >18 y; chief complaint of chest pain and/or shortness of breath; chest X-ray ordered by treating emergency physician | Referred with known diagnosis, diagnostic imaging known prior to data collection, pregnancy, unable to complete ultrasound, unable to consent due to clinical instability | 128 | 64 (NA) | 55 |
| Carlino MV 2018 | Italy | ED | 2017 | LUS, CXR | Admitted to ED for acute dyspnoea or worsening of chronic dyspnoea within the previous 48 h | Dyspnoea of traumatic origin | 100 | 72 (NA) | 56 |
| Chaitra S 2021 | Inda | ICU | 2017 | LUS | Age >18 y; admitted to ICU with acute respiratory failure: PaO₂ < 60 mmHg on room air; SpO₂ < 90% on room air; PaCO₂ > 50 mmHg with pH < 7.30 | Unusual etiology (e.g., chronic diffuse interstitial disease, fat embolism, tracheal stenosis, unidentified diagnosis post-hospitalisation, progression preventing conclusion, lung malignancy), intubation | 130 | 49 (15) | 64 |
| Chim E 2015 | USA | ED | 2009-2010 | LUS | Chief complain of dyspnoea | <18 years, incarcerated, pregnant, dialysis, non–English-speaking, dyspnoea was caused by another diagnosis (e.g., stab wound), chest radiograph already available and known to the sonographer, positive pressure ventilation, receiving nebuliser treatment, unable to consent | 380 | 55 (12) | 55 |
| Cibinel GA 2012 | Italy | ED | 2007 | LUS | Presenting to the ED with acute dyspnoea: sudden onset of dyspnoea without a history of chronic dyspnoea or worsening of chronic dyspnoea within 48 h | Experienced physician was not present, inability to consent | 56 | 82 (14) | 63 |
| Coiro S 2016 | Italy, France | Ward | 2014-2015 | LUS | Age >18 years; acute heart failure diagnosis per ESC criteria | Pulmonary fibrosis, significant pleural effusion, severe emphysema, pleurisy, pneumectomy or lobectomy, pulmonary cancer, breast prosthesis, pregnancy, planned revascularisation, long distance from hospital | 110 (94 analysed) | 73 (1) | 55 |
| De Carvalho H 2021 | France | ED | 2016-2018 | LUS | Age >75 y, acute respiratory failure (<24 h), RR ≥25/min, SpO₂ ≤95% on room air | Life expectancy <24 h, do-not-resuscitate order, immediate mechanical ventilation, no consent | 89 | 86 (5) | 38 |
| Dehbozorgi A 2019 | Iran | ED | 2016 | LUS | Age ≥18 y, undifferentiated acute dyspnoea | Chronic dyspnoea, ST-elevation MI, clear diagnosis after initial exam, prior HF treatment, refusal | 100 | 58 (12) | 47 |
| Dexheimer N 2015 | Brazil | ICU | 2011-2012 | LUS | Age ≥18 y, admitted to ICU for acute respiratory failure: RR ≥30, PaO₂ ≤60 mmHg, O₂ sat ≤90%, or PCO₂ ≥45 mmHg with pH ≤7.35 | Intubated before admission; multiple or rare diagnoses | 37 | 72 (15) | 43 |
| Elatroush HH 2023 | Egypt | ICU | 2017-2020 | LUS | Admitted with acute respiratory failure (e.g., ILD, IPF, acute pulmonary oedema, COPD exacerbation, pneumothorax, ARDS) | Age < 18 y, severe morbid obesity (BMI > 35 kg/m²) | 80 | 57 (13) | 53 |
| Elmahalawy II 2017 | Egypt | ICU | 2014-2015 | LUS, CXR | Age >18 y, ICU patients with chest problems in ICU, supported by clinical signs/symptoms or lab abnormalities (e.g., fever, cough, rales, elevated TLC, CRP) | Pregnant women, morbid obesity, untransferable patients | 130 | 43 (13) | 65 |
| Elnaem WH 2017 | Egypt | ICU | NA | LUS, CXR | Adults >18 y with new or recurrent dyspnoea | Age <18 or >70 years, trauma, high risk of PE (Wells Criteria), pregnancy | 90 | NA | NA |
| Farahmand S 2019 | Iran | ED | 2014-2015 | LUS | >18 y with acute dyspnoea at the ED | None | 120 | 61 (17) | 53 |
| Feuerstein D 2022 | Germany | ED | 2016 | LUS | >18 years presenting during the day with shortness of breath | NA | 99 | 62 (NA) | 58 |
| Gallard E 2015 | France | ED | 2012 | LUS | Age ≥18, nontraumatic acute dyspnoea, an ED physician trained in cardiopulmonary ultrasound was available | Prehospital care (except oxygen), needing ventilation, prior hospital care, no final diagnosis, non-dyspnoea admission, hospital stay <24h | 130 | 81 (NA) | 54 |
| Gargani L 2008 | Italy | Ward | 2004-2006 | LUS | Dyspnoea at admission (NYHA class II-IV), NT-proBNP at admission, ULC assessment within 4h of NT-proBNP, no diuretics between the two assessments | Time delay between measurements, missing lab values, sonography unavailable, failure to notify sonographer | 149 | 71 (NA) | 66 |
| Ghauri S 2021 | Pakistan | ED | 2018-2019 | LUS, CXR | Age ≥18 y, both genders, acute dyspnoea on arrival at ED, respiratory rate >30 breaths/min | Pregnancy, previous diagnosis of acute pulmonary ooedema, acute chest wall injury | 225 | NA | NA |
| Glöckner E 2020 | Germany | ED | 2014-2015 | LUS | ≥18 y, presenting to ED with undifferentiated acute dyspnoea | Pleural effusion >2 quadrants, pneumothorax, lack of lung sliding, ILD, pneumonia, prior lung resection, lung cancer, mechanical ventilation, pleural drainage | 89 | 73 (60-80) | 58 |
| Heldeweg MLA 2022 | The Netherlands | ED | 2018-2020 | LUS | >18 y admitted to ICU with clinically indicated lung ultrasound | Pneumothorax; simultaneous occurrence of CPE and NCIS | 101 | 64 (15) | 68 |
| Hubert A 2019 | France | Ward | 2016-2017 | LUS | ≥18 y old, dyspnoea (NYHA≥2) underwent a coronary angiography with left heart catheterisation | Mitral stenosis, pulmonary fibrosis, pneumonia, active lung cancer, recent chest trauma. Change in diuretic treatment between TTE and LHC | 81 | 75 (67-80) | 68 |
| Johannessen Ø 2023 | Norway | Ward | 2017, 2020-2021 | LUS | Age ≥18 y, hospitalised with acute exacerbation of COPD, diagnosed by attending physician based on GOLD criteria, consent obtained. | Pneumothorax, thoracic deformities, recent thoracic surgery, asthma, pulmonary fibrosis, pleural disease, COVID-19, mental disorientation, dementia, or inability to consent. | 123 | 75 (9) | 46 |
| Joseph RB 2024 | India | ICU | 2020-2021 | LUS, CXR | ≥18 y with acute respiratory failure, underwent LUS and chest X-ray | History of lung cancer, prior lung surgery, interstitial lung fibrosis | 100 | 54 (12) | 64 |
| Koh Y 2018 | Singapore | ED | 2015 | LUS | ≥21 y old, able to provide informed consent, presenting with dyspnoea | Refused consent or dyspnoea primarily exertional in nature | 231 (199 analysed) | 68 (16) | 63 |
| Lajili F 2024 | Tunisia | ED | 2022-2023 | LUS | ≥18 y, presenting to ED with acute exacerbation of COPD | Hemodynamic or respiratory instable, altered consciousness (GCS ≤13), trauma-related, no consent | 380 | 68 (11) | 66 |
| Laursen CB 2016 | Denmark | Prehospital | 2013-2014 | LUS | Respiratory rate >30 breaths/min or oxygen saturation <90% without supplemental oxygen | Age <18 years, permanent mental disability, or PLUS delaying life-saving treatment/transport | 40 | 74 [67-81] | 35 |
| Lichtensten DA 2008 | France | ICU | NA | LUS | 22-91 y admitted to the ICU with acute respiratory failure based on clinical and biological criteria | No final diagnosis at discharge; multiple concurrent diagnoses; rare causes of dyspnoea with frequency < 2% (e.g., fat embolism, massive pleural effusion, tracheal stenosis, chronic interstitial disease) | 260 | 68 (16) | 54 |
| Lichtensten DA 2009 | France | ICU | NA | LUS | Mechanically ventilated critically ill patients undergoing pulmonary artery catheterisation | Hindrances to ultrasound exam (e.g. pneumothorax, extensive dressings) | 102 | 57 (19) | 61 |
| Liteplo AS 2009 | USA | ED | 2006-2007 | LUS | ≥18 y, dyspnoea, NT-proBNP ordered as part of clinical workup | Inability to consent or not having an NT-ProBNP level sent. | 94 | 74 (14) | 59 |
| Liu ZP 2016 | China | ED | 2014-2015 | LUS | Admitted to the ED with acute dyspnoea | Rapid arrhythmia, severe bradycardia, conduction disturbance, ACS, PE, hypertensive crisis, tamponade, peripartum cardiomyopathy, anemia, kidney dysfunction, thyroid disease, iatrogenic causes | 58 | 77 (10) | 60 |
| Mayr U 2022 | Germany | ICU | 2017-2018 | LUS | Critically ill patients with hemodynamic monitoring via TPTD on ICU admission | Pleural effusion in scanning regions, pulmonary vascular occlusion, major unilateral pathologies (e.g., pleural effusion, pneumothorax, thoracic drainage, atelectasis, tumorous lesion, lung resection) | 50 | 65 [55-72] | 64 |
| Miger K 2019 | Denmark | ED | 2017-2018 | LUS | Age ≥50 y, acute dyspnoea, ≥1: RR >20/min, SpO₂ <95% (<92% if COPD), O₂ need, signs of HF (bilateral rales, oedema, JVD), rhonchi, prolonged expiration | ICU/inotropes/ventilation need, ACS, dementia/language barrier, not Danish citisen, life expectancy <3 months, refusal, CT already indicated for PE/aortic disease, CT/echo not possible within 12h | 117 | 74 (NA) | 45 |
| Modi UP 2024 | India | ED | 2024 | LUS | ≥18 y, presenting with respiratory complaints or signs of respiratory distress, patient/relatives consent | Pregnancy, COVID-19 positive | 150 | NA | 63 |
| Msolli MA 2021 | Tunisia | ED | 2016-2017 | LUS | ≥ 18 y, admitted to ED for acute dyspnoea | No consent, post-traumatic dyspnoea, pregnancy, intubation, inotropic drugs, instability, protocol violation | 700 | 68 (20) | 59 |
| Mumoli N 2016 | Italy | ED, ward | 2014 | LUS | Admitted to ED for dyspnoea (RR > 24/min, O2 sat <92% or oxygen therapy), hospital admission planned | Age <18 y, trauma, known pneumothorax, dialysis or intensive observation, severe instability of vital signs, no consent, mental disability | 226 | 79 (13) | 42 |
| Nagaharu K 2022 | Japan | ED | 2017-2018 | LUS | Admitted to the ED for dyspnoea, care by participating physician | Age <18 y, no consent, requiring urgent interventions | 95 | 82 (NA) | 59 |
| Nakao S 2021 | Canada | ED | 2017 | LUS, CXR | ≥50 y, dyspnoea or cough, suspected acute HF or COPD, LUS performed | STEMI on ECG, interstitial fibrosis, extensive lung cancer, pneumonectomy/lobectomy, pneumothorax | 81 | 79 [73-86] | 49 |
| Neeraj G 2023 | ICU | India | 2020-2021 | LUS | >18 y, admitted to ICU with dyspnoea | No consent from patients/relatives | 150 | NA | 65 |
| Ocel S 2024 | ED | Turkey | NA | LUS | Admitted to ED with respiratory distress | ≤18 y, USG could not be performed, respiratory distress due to non-respiratory causes | 120 | 68 [59-77] | 64 |
| Oehman J 2019 | ED | Finland | 2014-2015 | LUS, CXR | Presenting to ED with dyspnoea | Unsatisfactory ultrasound visibility, pulmonary fibrosis, age <18 y, mitral stenosis, prosthetic mitral valve | 100 | 71 (15) | NA |
| Patel CJ 2018 | ED, ICU | India | NA | LUS | >12 y; informed consent; acute respiratory distress requiring ICU admission | Prior diagnosis, lack of consent | 50 | 60 (NA) | 64 |
| Perrone T 2017 | Ward | Italy | 2014-2016 | LUS, CXR | Admitted to Internal-Medicine wards with an ED diagnosis of “dyspnoea.” | Pulmonary cancer, fibrothorax, congenital lung disease, severe chest deformities, recent chest surgery | 130 | 81 (9) | 46 |
| Pivetta E 2015 | ED | Italy | 2010-2012 | LUS, CXR | >18 y, presenting to the ED with acute dyspnoea | Dyspnoea due to obvious non-ADHF causes, invasively ventilated | 1005 | 77 (13) | 54 |
| Prosen G 2011 | Prehospital | Slovenia | 2007-2010 | LUS | Dyspnoea as primary complaint: sudden onset without prior history of chronic dyspnoea or an increase in severity of chronic dyspnoea | <18 y, renal insufficiency, trauma, severe coronary ischemia, other causes of dyspnoea (pneumonia, pulmonary embolism, carcinoma, pneumothorax, pleural effusion, intoxication, anaphylaxis, airway obstruction, bronchial stenosis, GERD) | 218 | 63 (NA) | 71 |
| Russell F 2017 | ED | USA | 2012-2013 | LUS | >18 y, undifferentiated dyspnoea (≥2 possible etiologies) | Clinician confident of diagnosis after initial assessment, STEMI on ECG, treated for ADHF >30 min before US, refused consent, previously enrolled | 99 | 56 (13) | 56 |
| Russell F 2023 | Prehospital | USA | 2022 | LUS | >18 y, dyspnoea, ≥1: bilateral leg oedema, orthopnoea, wheezing or rales on auscultation, respiratory rate >20, or O2 saturation <92% | SBP <90 mmHg, STEMI on ECG, fever >100.4°F, trauma, pregnancy, transport to hospital without accessible EMR | 264 | 65 (NA) | 43 |
| Saboo R 2022 | ICU | India | 2019-2021 | LUS | Presenting with acute dyspnoea requiring ventilatory support | NA | 96 | 55 (17) | 43 |
| Sartini S 2016 | ED | Italy | 2011-2013 | LUS, CXR | ≥18 y, acute dyspnoea (<24h onset or worsening over 4 days), non-trauma | None | 236 | 80 (12) | 50 |
| Schoeneck JH 2021 | Prehospital | USA | NA | LUS | ≥18 y, chief complaint of dyspnoea ≥1 of: RR >20, SpO₂ <92%, abnormal lung sounds, increased work of breathing, history of orthopnoea/pedal oedema | NA | 65 | 64 (17) | 57 |
| Secko M 2022 | ED | USA | 2018-2019 | LUS | Hemodynamically stable, presenting to ED with chief complaint of dyspnoea | <18 y, requiring life-saving procedures, unable to consent without legally authorised representative present | 102 | 63 (19) | 56 |
| Seibel A 2020 | ICU | Germany, Austria | 2013-2015 | LUS | ICU patients requiring advanced hemodynamic monitoring by transpulmonary thermodilution | <18 y; pneumothorax | 184 | 66 [NA] | 69 |
| Sekiguchi H 2015 | ICU | USA | 2010 | LUS | Adults, admitted to ICU with acute hypoxemic respiratory failure or PaO₂/FiO₂ < 300 within 6 h of diagnosis or ICU admission | ICU provider declined ultrasound, exam interfered with patient care, no sonographer available within 6 h | 134 | 68 [55-77] | 59 |
| Sen S 2017 | Ward | USA | 2014-2015 | LUS | >18 y, hospitalised on general wards, Medical Emergency Team activation for respiratory deterioration | Pregnant patients, patients in cardiac arrest, MET activation in ICU or ED | 50 | 69 (13) | 60 |
| Sforza A 2017 | ED | Italy | 2014-2015 | LUS, CXR | Presenting to ED with acute dyspnoea or worsening of chronic dyspnoea within previous 48 h | NA | 68 | 78 (12) | 62 |
| Sharma A 2025 | ED, ICU | India | 2023 | LUS | Acute dyspnoea, including new onset or exacerbation | <18 y, trauma, CKD, ACS (unless primary complaint was dyspnoea), other causes (e.g., PE, pneumonia, COPD) | 104 | 55 (NA) | 60 |
| Siddiqi M 2025 | ED | Pakistan | 2022-2024 | LUS | 20–60 y, presenting with sudden onset dyspnoea | Chest trauma, foreign body inhalation, lung/pleural malignancy, severe ascites | 219 | 35 (11) | 63 |
| Tsverava M 2010 | Ward | Georgia | 2004-2009 | LUS | Patients admitted for worsening HF | NA | 380 | 63 (11) | 60 |
| Vauthier C 2021 | ED | France | 2020 | LUS | ≥18 y, acute dyspnoea, cardiopulmonary ultrasound done | Pregnancy | 103 | 73 (17) | 50 |
| Vitturi N 2011 | Ward | Italy | 2007-2008 | LUS, CXR | Admitted with dyspnoea | NA | 152 | NA | NA |
| Volpicelli G 2014 | ICU | Italy | 2008-2010 | LUS | Critically ill ICU patients requiring invasive hemodynamic measurements | Unable to perform heart/lung ultrasound (e.g., extensive dressings, pneumothorax, fibrothorax, pneumonectomy) | 73  (32 analysed) | 62 (15) | 68 |
| Wooten WM 2018 | ED | USA | 2016-2017 | LUS, CXR | Admitted due to dyspnoea | No consent | 99 | 67 (14) | 51 |
| Yang F 2017 | Ward | China | 2016 | LUS | >18 y; ADHF with NYHA class III–IV per 2016 ESC guidelines | Pulmonary interstitial disease (fibrosis, pneumonia, severe pneumonitis) ARDS, congenital heart and valve diseases | 82 | 70 (16) | 65 |
| Zhong L 2012 | ED | China | 2010-2012 | LUS | Presented to ED with dyspnoea (including those with sudden onset of dyspnoea or worsening of underlying chronic illness) | <18 y; renal insufficiency, severe coronary artery disease, moderate to large pericardial or pleural effusions, upper airway obstruction, pneumonia, pneumothorax, or pulmonary embolism | 58 | 68 (NA) | 60 |
| Zhou S 2014 | ICU | China | 2012-2013 | LUS, CXR | 14–90 y; suspected AECOPD; admitted to ICU within 24 h; no thoracic trauma or deformity, informed consent obtained | History of chest trauma, poor acoustic window | 89 | NA | NA |
| Zhu L 2024 | Ward | China | 2014-2015 | LUS | Suspected cardiac origin respiratory distress, able to cooperate to ultrasound | Concomitant diseases involving other systems than cardiopulmonary, pulmonary tumors, trauma, obesity leading to blurred ultrasound images | 170 | NA | NA |
| Supplemental Table 1d. Study characterisations for chest radiography studies | | | | | | | | | |
| Ely W 2001 | USA | ICU | 1997 | CXR | Adults in ICU with pulmonary artery catheter scheduled for a digital CXR | Recent cardiothoracic surgery or chest trauma | 100 | 59 (NA) | 52 |
| Gillespie ND 1997 | UK | ED | NA | CXR | Admitted with acute dyspnoea | Isolated pneumothorax, pneumonia, pulmonary emboli, cor pulmonale, renal failure | 71 | 73 (NA) | 42 |
| Kennedy S 2011 | USA | ED | 2005 | CXR | BNP ≥ 300 pg/mL, ejection fraction ≤ 30%, admission and discharge diagnosis of CHF. Control group: no CHF, chest X-ray available. | NA | 55 | NA | NA |
| Knudsen CW 2004 | USA, Norway, France | ED | 1999-2000 | CXR | Presenting with acute dyspnoea, sudden onset or worsening of chronic dyspnoea | Obvious non-HF causes of dyspnoea, MI or advanced renal failure, incomplete data | 880 | 64 (16) | 55 |
| Martinez-Rumayor AA 2010 | USA | ED | NA | CXR | ≥21 y, presentation with chief complaint of dyspnoea | Severe renal insufficiency (creatinine >2.5 mg/dL), dyspnoea from trauma, severe ischemia, >2h delay after IV diuretics, unblinded NT-proBNP | 599 | NA | 51 |
| Mueller-Lenke N 2006 | Switzerland | ED | NA | CXR | Acute dyspneic patients from the BASEL study who had a chest X-ray in the ED before or immediately after IV diuretics or vasodilators, with radiologist interpretation. | Patients with external chest radiograph before ED visit, no CXR in ED, or no formal radiological reading | 202 | NA | NA |
| Panda R 2023 | India | ICU | 2020-2021 | CXR | >18 y, dyspnoea; ICU stay ≥24 h | Pregnant, incomplete or uninterpretable data | 174 | 53 [NA] | 62 |
| Studler U 2008 | Switzerland | ED | 2001-2002 | CXR | Presenting with dyspnoea to ED | Chest radiographs taken in seated position; radiographs no longer available due to no PACS | 277 | 72 (15) | 58 |
| Tækker M 2022 | Denmark | ED | 2019 | CXR | Non-traumatic patients, ≥40 y, admitted to the ED and referred for supine chest X-ray (sCXR) | No informed consent, participation would delay life-saving treatment, >4-hour interval between sCXR and CT, enrolled twice | 91 | 78 (11) | 47 |

ACS: Acute Coronary Syndrome, ADHF: Acute Decompensated Heart Failure, AECOPD: Acute Exacerbation of Chronic Obstructive Pulmonary Disease, ARDS: Acute Respiratory Distress Syndrome, BMI: Body Mass Index, BNP: B-type Natriuretic Peptide, CAP: Community-Acquired Pneumonia, CHF: Congestive Heart Failure, CKD: Chronic Kidney Disease, COPD: Chronic Obstructive Pulmonary Disease, CPE: Cardiogenic Pulmonary Oedema, CRP: C-reactive Protein, CT: Computed Tomography, CXR: Chest X-Ray, EF: Ejection Fraction, EMR: Electronic Medical Record, ESC: European Society of Cardiology, FiO₂: Fraction of Inspired Oxygen, GCS: Glasgow Coma Scale, GERD: Gastroesophageal Reflux Disease, HF: Heart Failure, ICU: Intensive Care Unit, ILD: Interstitial Lung Disease, IPF: Idiopathic Pulmonary Fibrosis, IV: Intravenous, JVD: Jugular Venous Distention, LHC: Left Heart Catheterisation, LUS: Lung Ultrasound, LV: Left Ventricle, MI: Myocardial Infarction, NT-proBNP: N-terminal pro-B-type Natriuretic Peptide, NYHA: New York Heart Association, O₂ sat / SpO₂: Oxygen Saturation, PaCO₂: Partial Pressure of Carbon Dioxide (arterial), PaO₂: Partial Pressure of Oxygen (arterial), PE: Pulmonary Embolism, RHC: Right Heart Catheterisation, ReDS: Remote Dielectric Sensing, RR: Respiratory Rate, SBP: Systolic Blood Pressure, STEMI: ST-Elevation Myocardial Infarction, TLC: Total Leukocyte Count, TPTD: Transpulmonary Thermodilution, ULC: Ultrasound Lung Comets, US / USG: Ultrasound, NA: Not Available

| Supplemental Table 2. Study information on index test and reference | | | | | | |
| --- | --- | --- | --- | --- | --- | --- |
| Study | **Index test(s)** | **Index test description** | **Hours from admission to test** | **Reference** | **Reference description** | **Prevalence (%)** |
| Aggarwal M 2016 | LUS | 28-zones; positive if ≥3 B-lines <7 mm apart seen bilaterally. | NA | ADHF | Clinical evaluation, medical records, chest X-ray, ECG, BNP > 266 pg/mL, echocardiography. Final diagnosis by the treating physician, blinded to lung ultrasound but had access to all other diagnostic tests. | 81 |
|  | CXR | Posteroanterior and lateral | NA |  |  |  |
| Ali M 2022 | LUS | Supine or semi-setting position, curvilinear and linear probes. Pulmonary oedema if ≥3 bilateral B-lines with or without pleural effusion. | NA | Pulmonary oedema | Chest CT scan | 24 |
|  | CXR | Supine chest X-ray (standard hospital protocol) | NA |  |  |  |
| Anderson KL 2013 | LUS | 8-zones; ≥2 bilateral zones with ≥3 B-lines = positive (Volpicelli BBPC method) | NA | ADHF | Diagnosis based on post-discharge chart review (ED note, labs incl. BNP, radiology, echo, discharge summary). To physicians, in disagreement a third. | 44 |
| Arthur M 2023 | LUS | 14-zones; semirecumbent, 1-5 MHz | < 24 | Pulmonary oedema | Composite reference standard (clinical data, labs, imaging including CXR ± CT, and final discharge diagnosis) | NA |
| Azab NY 2022 | LUS | Bedside Lung Ultrasonography in Emergency Department protocol (BLUE) | NA | Pulmonary oedema | Chest CT scan | 14 |
|  | CXR | Standard postero-anterior (upright) portable CXR | NA |  |  |  |
| Baid H 2022 | LUS | BLUE protocol, supine position. Pulmonary oedema defined as bilateral diffuse alveolar syndrome (multiple B-lines throughout) | 0.27 | Pulmonary oedema | Final composite diagnosis formulated by two Emergency Medicine consultants, with access to the history, examination, and investigations performed during the ED stay. Up to four concurrent diagnoses were permitted to be made for each patient. | 15 |
| Baker K 2016 | LUS | 8-zones; 2–5 MHz curved probe (GE Logic-e). Positive scan: ≥3 B-lines in ≥2 zones bilaterally | NA | Pulmonary oedema | Post-discharge chart review by an expert cardiologist, assessing all clinical notes, tests, imaging (excluding LUS) to determine if the patient’s breathlessness was due to pulmonary ooedema or not. | 20 |
| Baker K 2016 | CXR | Standard chest x-ray | NA | Pulmonary oedema | Post-discharge chart review by an expert cardiologist, assessing all clinical notes, tests, imaging (excluding LUS) to determine if the patient’s breathlessness was due to pulmonary ooedema or not. | 20 |
| Baldi G 2013 | LUS | 28 zones; interstitial syndrome was defined as a B-line score >5. | NA | Pulmonary oedema | Quantitative lung CT scan used to determine lung weight and density as a surrogate for extravascular lung water | 71 |
| Balen F 2021 | LUS | 10-zones; B-profile suggestive of AHF | NA | AHF | Final diagnosis by 2 experts (pneumologist, cardiologist), blinded to index test, using patient chart including clinical course, labs, imaging | 66 |
| Bataille B 2014 | LUS | 6 zones; anterior and lateral chest zones examined. AIS > 2 B-lines in a region (B-profile) | NA | Pulmonary oedema | Final diagnosis by two independent experts, based on laboratory tests, including troponin I and B-type natriuretic peptide, chest radiography, high-resolution CT imaging (55%) and transthoracic Doppler echocardiography by a senior cardiologist (26%) | 25 |
| Bekgoz 2019 | LUS | 6-zones; BLUE protocol, a convex probe (3.5–5 MHz). Positive B-profile = bilateral anterior lung fields with ≥3 B-lines. | 0.33 | Pulmonary oedema | Diagnosis was made by treating physicians based on the clinical course. Access to chest X-ray, echocardiography by a cardiologist, thoracic CT, respiratory function tests, lower extremity venous Doppler ultrasonography, laboratory results, treatment response | 25 |
| Bitar Z 2015 | LUS | 8-zones (4 anterior, 4 lateral); B-profile defined as 3 or more B-lines in ≥1 scanning zone | NA | NT-proBNP | NT-proBNP thresholds: ≥450 pg/mL (<50 yrs), ≥900 pg/mL (50–75 yrs), ≥1800 pg/mL (>75 yrs) used as reference standard for acute heart failure | 82 |
| Bouzidi H 2025 | LUS | 8-zonea; ≥3 B-lines in ≥2 zones bilaterally | < 2 | AHF | Transthoracic echocardiography used as the reference standard for diagnosis of AHF in patients presenting with acute dyspnoea | 79 |
|  | CXR | NA | NA |  |  |  |
| Buessler A 2019 | LUS | 8-zones; a positive zone was defined as the presence of at least 3 B-lines. | NA | AHF | Final diagnosis at discharge collected from medical records, adjudicated by 2 senior physicians blinded to the LUS measurements | 62 |
| Buhumaid RE 2019 | LUS | 6-zones (bilaterally anterior, lateral, posterior); Pulmonary oedema defined as presence of diffuse bilateral B-lines (≥3 lines per zone), with or without pleural effusion. | NA | Pulmonary oedema | Composite clinical diagnosis determined by treating physicians based on overall clinical evaluation, available imaging modalities (including chest X-ray, CT), laboratory results, and clinical judgment | 15 |
|  | CXR | Routine, reviewed by radiologist. | NA |  |  |  |
| Carlino MV 2018 | LUS | 4-zones: 2 zones per hemithorax. Positive if bilateral interstitial syndrome (≥3 B-lines per zone) and/or pleural effusion was present in the lateral zones bilaterally. | < 0.5 | AHF | Diagnosis based on chart review from ED admission to discharge by two independent observers (emergency physician & internal medicine specialist), using the following investigations: Medical history, Clinical examination, Blood gas analysis, Chest X-ray, Electrocardiogram, Routine blood tests (including NT-proBNP) | 39 |
|  | CXR | NA | NA |  |  |  |
| Chaitra S 2021 | LUS | 3 zones BLUE protocol (anterior, lateral, posterolateral); profiles: A, B, B’, C, A/B, PLAPS; pulmonary oedema defined as bilateral 3 B-lines with or without lung sliding |  | Pulmonary oedema | Final diagnosis established by clinical and radiological (CT) examinations, lab data (e.g., infection profile), and functional testing (e.g., COPD) | 22 |
| Chim E 2015 | LUS | 8-zones; anterior and lateral zones (superior/inferior bilaterally). Positive if ≥3 B-lines in 2 or more zones bilaterally. | NA | AHF | Two independent experts (EP and cardiologist) reviewed abstracted chart data to adjudicate AHFS as primary cause of dyspnoea | 35 |
| Cibinel GA 2012 | LUS | 8-zones (bilateral anterior and lateral); AIS defined as ≥2 regions bilaterally with ≥3 B-lines per zone | 0.5 | Cardiogenic dyspnoea | Final diagnosis made by an expert emergency physician and a cardiologist based on full chart review including labs, imaging, echo, and discharge summary, blinded to ultrasound (PLUS) results | 48 |
| Coiro S 2016 | LUS | 28-zones; scanned 2nd–4th (5th on right) ICS along 4 lines (parasternal, midclavicular, anterior & midaxillary); B-lines total score 0–280. 21 B-lines used as cut off according to Youden. | NA | BNP | >400 pg/mL | 69 |
| De Carvalho H 2021 | LUS | 12 zones; 6 quadrants in each hemithorax. Profiles (A, AB, B, C, PLAPS) were used based on the worst findings in each quadrant. Classification based on Gallard et al. and ASE guidelines. | NA | Pulmonary oedema | Diagnosis by adjudication committee based on full clinical data, lab tests, imaging, and chest ultrasound raw data | 48 |
| Dehbozorgi A 2019 | LUS | 8 zones (anterior/lateral); B-lines defined as ≥3 vertical artifacts per zone. B-lines considered positive for AHF if at least one B-profile is present bilaterally | 0.5 | AHF | Final diagnosis of acute heart failure AHF made by two independent specialists based on post-discharge patient records (labs, imaging, notes). | 28 |
| Dexheimer N 2015 | LUS | Bilateral B-profile defined as diffuse anterior bilateral B-lines with lung sliding; number of zones not specified. | 0.33 | Pulmonary oedema | Final diagnosis of acute heart failure as cause of ARF, made by independent experts after full chart review | 41 |
| Elatroush HH 2023 | LUS | BLUE protocol; scanning anterior, lateral, posterior axillary regions; positive for pulmonary oedema = lung sliding + multiple B-lines. | NA | Pulmonary oedema | Chest CT scan | 4 |
| Elmahalawy II 2017 | LUS | 12-zones; 5–9 MHz micro convex probe, patient in supine position, anterior and lateral views assessed. LUS positive for if multiple B-lines were present | NA | Pulmonary oedema | Chest CT was performed in the supine position from lung apex to base. CT scans were evaluated for mediastinal, pleural, and parenchymal pathology using standardised anatomical landmarks and definitions from the Fleischner Society. | 15 |
|  | CXR | Anteroposterior portable CXR in supine position, reviewed by radiologist. | NA |  |  |  |
| Elnaem WH 2017 | LUS | 18-zones (9 zones per hemithorax: 4 anterior, 2 lateral, 3 posterior); positive test for pulmonary oedema = ≥3 B-lines per zone | < 6 | Pulmonary oedema | Thoracic CT was performed in all patients using a multi-detector CT scanner; used as the gold standard to confirm pulmonary oedema, pneumothorax, pleural effusion, and alveolar consolidation, evaluated by an expert radiologist | 33 |
|  | CXR | Anteroposterior supine chest radiograph performed using portable X-ray unit | < 6 |  |  |  |
| Ely W 2001 | CXR | Anteroposterior, supine digital chest X-ray | NA | PAOP | Pulmonary artery occlusion pressure measured via Swan-Ganz catheter; threshold ≥18 mm Hg used as surrogate for hydrostatic pulmonary oedema | 39 |
| Farahmand S 2019 | LUS | 8 zones; bilateral B-pattern count ≥ 2 zones; per Volpicelli et al. | NA | BNP | BNP level >500 pg/ml used as the diagnostic standard for ADHF | 48 |
| Feuerstein D 2022 | LUS | Interstitial syndrome defined as ≥3 B-lines bilaterally per intercostal space. | NA | ADHF | Discharge diagnosis based on standard diagnostic work-up including clinical assessment, radiologic imaging (CXR/CT), and/or NT-proBNP. | 24 |
| Gallard E 2015 | LUS | 6-zones (anterior, lateral, and posterior); B-lines defined as ≥3 comet-tail artifacts per intercostal space | NA | Acute left-sided heart failure | Final diagnosis by two independent physicians using full clinical data incl. lab, CXR, echo, and NT-proBNP | 62 |
| Gargani L 2008 | LUS | 28-zones; parasternal/midclavicular/anterior & mid-axillary lines; ULC score >5 = positive | NA | Cardiogenic dyspnoea | Diagnosis of acute cardiogenic dyspnoea vs non-cardiogenic causes based on full clinical workup including imaging, labs, and clinical follow-up | 82 |
| Ghauri S 2021 | LUS | 8 zones; Presence of comet sign (≥3 B-lines) in ≥2 quadrants considered diagnostic for APE | NA | Pulmonary oedema | Clinical diagnosis based on presence of all: raised jugular venous pressure, pedal ooedema, S3 on cardiac auscultation, and bilateral crepitations on lung auscultation | 55 |
|  | CXR | Presence of bilateral infiltrates on chest X-ray considered diagnostic for APE | NA |  |  |  |
| Gillespie ND 1997 | CXR | NA | NA | Left ventricular systolic dysfunction | Echocardiographic assessment of LVEF based on fractional shortening <20%, regional wall motion abnormalities, or dilated LV on M-mode | 63 |
| Glöckner E 2020 | LUS | 8-zones; supine, 6-sec clips, 2–5 MHz. Positive if ≥2 bilateral regions had ≥3 B-lines. | < 1 | AHF | Adjudicated diagnosis based on available clinical, lab, imaging and follow-up data. Determined by two independent experts (cardiologist and emergency physician); disagreements resolved by a third. | 54 |
| Heldeweg MLA 2022 | LUS | 6-zones; BLUE protocol. Interstitial syndrome was defined by the presence of a B-pattern (≥3 B-lines in one frame), bilateral | NA | Pulmonary oedema | Post-hoc expert adjudication of clinical diagnosis based on full chart review (including imaging, labs, physical exam) but excluding lung ultrasound findings. | 27 |
| Hubert A 2019 | LUS | 8-zones (4 zones per hemithorax); B-lines counted 0–10 per zone; total LUS score 0–80; dichotomized with cut-off ≥3 | < 2 | LVEDP | Invasive left heart catheterisation measuring left ventricular end-diastolic pressure, positive >= 20 mmHg | 35 |
| Imamura T 2022 | ReDS | Positive >28% lung fluid content according to Youdens index | NA | PCWP | Pulmonary capillary wedge pressure >15 mmHg measured via right heart catheterisation | NA |
| Inui G 2024 | CT | Supine chest CT, used sign of ostial pulmonary vein >12.5 mm. | < 24 | Pulmonary oedema | Clinical diagnosis based on symptoms, physical examination, laboratory values including BNP, and visual assessment of chest CT | 74 |
| Izumida T 2022 | ReDS | Positive >26.5% lung fluid content according to Youdens index | NA | BNP | BNP >100 pg/mL | 71 |
| Izumida T 2023 | ReDS | >35% lung fluid content positive for pulmonary congestion | NA | Pulmonary congestion | Lung ultrasound: 8-zone, sitting position, positive if bilateral three or more B-lines at each zone | 5 |
| Johannessen Ø 2023 | LUS | 8-zones; positive LUS defined as ≥3 B-lines in ≥2 zones bilaterally | < 24 | AHF | Adjudicated by three cardiologists blinded to LUS. Patient records (charts, labs, imaging, ECG, echocardiograms) were reviewed, and diagnoses were based on ESC criteria incorporating signs, symptoms, and objective myocardial abnormalities. HF was classified as concurrent/contributing or non-concurrent to the AECOPD. | 39 |
| Joseph RB 2024 | LUS | NA | NA | Pulmonary oedema | Final diagnosis determined by senior critical care team with clinical examination and CT scan when available | 19 |
|  | CXR | NA | NA |  |  |  |
| Kennedy S 2011 | CXR | NA | NA | CHF | Admission and discharge diagnosis and BNP ≥300 pg/mL, EF ≤30%. | 44 |
| Knudsen CW 2004 | CXR | NA | NA | AHF | Case report forms, ECGs, chest X-rays, echocardiograms, test results, consultations, and chart data were reviewed by two independent cardiologists; disagreements were resolved by the study endpoint committee. | 51 |
| Koh Y 2018 | LUS | 6 zones; modified BLUE protocol (mid-clavicular, anterior axillary, paravertebral/posterior axillary); B-profile defined as ≥3 B-lines bilaterally | < 4 | Pulmonary oedema | Final adjudicated diagnosis by two independent emergency physicians, based on history, clinical, laboratory, and radiological findings | 42 |
| Lajili F 2024 | LUS | 8-zones (anterior and lateral chest, right and left, upper and lower); The B-lines score suggestive of CHF ≥15. | < 4 | AHF | The final leading diagnosis of dyspnoea was determined by two independent senior EM physicians based on clinical presentation, exam findings, and diagnostic tests (CXR, echocardiography, BNP); disagreements were resolved by a third senior EM physician. | 41 |
| Laursen CB 2016 | LUS | 4-zones: 2 anterior and 2 lateral zones. ISS >=3 B-lines in a longitudinal plane between two ribs in ≥2 scanning fields bilaterally. | NA | Pulmonary oedema | One cardiologist and one emergency physician independently reviewed each hospital stay. Diagnosis required at least two of the following: clinical signs of pulmonary oedema (e.g., tachypnea, hypoxemia, bilateral crackles), imaging evidence (CXR, CT, or in-hospital LUS), or an underlying condition causing pulmonary oedema (e.g., systolic/non-systolic HF, ACS, hypertensive crisis, arrhythmia, valvular disease). Disagreements were resolved by a third physician. | 45 |
| Lichtensten DA 2008 | LUS | 6-zones; BLUE protocol (anterior, lateral, PLAPS); ISS = ≥3 B-lines per view; profiles defined (A, B, etc.) | NA | Pulmonary oedema | Diagnosis based on history, clinical exam, chest radiograph interpreted by radiologist, echocardiography, and response to treatment following American Heart Association criteria | 25 |
| Lichtensten DA 2009 | LUS | Anterior chest; bilateral B-lines (lung rockets) = B-predominance; absence = A-predominance | NA | PAOH | Pulmonary artery occlusion pressure measured via pulmonary artery catheter (Swan-Ganz) <= 13 mmHg | 60 |
| Liteplo AS 2009 | LUS | 8-zones; ISS = ≥3 B-lines per zone; positive = ≥2 zones bilat. | < 12 | CHF | Consensus diagnosis from chart review (labs, echo, CXR, discharge summary, etc.) by two EPs, with third reviewer as tiebreaker if needed. | 43 |
| Liu ZP 2016 | LUS | 4-zones; BLUE protocol points (upper and lower BLUE points bilaterally). B-lines scored 0–4 based on number: 0–3 = 1; 3–6 = 2; 6–8 = 3; ≥8 = 4. Total B-line score calculated by summing each point. Supine or semi-recumbent position. Optimal cut-off 8 B-lines | < 0.5 | Acute left ventricular heart failure | Final clinical diagnosis determined by three experienced ED physicians (≥6 years of experience), based on clinical assessment, labs, and imaging. | 47 |
| Martinez-Rumayor AA 2010 | CXR | NA | NA | AHF | Final diagnosis assigned by two independent cardiologists using full clinical data (including radiographic results but excluding NT-proBNP); disagreements resolved using Framingham criteria. | 45 |
| Mayr U 2022 | LUS | 28-zones; antero-lateral scan (14 intercostal spaces bilaterally); ISS = summed B-lines count per zone, cut off > 17 | NA | EVLW | Measured using transpulmonary thermodilution via PiCCO-2 device; indexation to predicted body weight; triple saline bolus injections via jugular CVC, pulmonary oedema if >= 10 | NA |
| Miger K 2019 | LUS | 14-zones; 2.5-3.5 MHz cardiac probe, 8 anterior, 6 posterior zones. Positive: ≥3 B-lines bilaterally and/or bilateral pleural effusion | < 6 | Pulmonary congestion | Radiologists evaluated pulmonary congestion on LDCT. Blinded to each other and all clinical data. | 27 |
| Miger K 2024 | CT | Non-contrast low-dose chest CT | 2.5 | AHF | Diagnosis of AHF based on clinical evaluation, echocardiography, labs, and consensus by expert panel. | 28 |
|  | CXR | Anterior-posterior or posterior-anterior (standard chest X-ray) | 2.5 |  |  |  |
| Modi UP 2024 | LUS | 6-zones; BLUE protocol (upper BLUE point, lower BLUE point, PLAPS point); diagnosis based on B profile (diffuse bilateral anterior B+ lines with lung sliding) | NA | Pulmonary oedema | Diagnosis established clinically in the ED by treating physicians, based on history, physical examination, imaging (CXR/LUS/echo where indicated), and response to therapy. No single uniform test applied as gold standard. | 30 |
| Msolli MA 2021 | LUS | 8-zones, BLUE protocol; B-profile defined as ≥3 B-lines in 2 anterior zones bilaterally | < 4 | AHF | Final diagnosis at hospital discharge, based on clinical findings, chest X-ray, brain natriuretic peptide, cardiac and lung ultrasound testing. | 53 |
| Mueller-Lenke N 2006 | CXR | CXR performed in ED; many patients required supine position. | NA | CHF | Discharge diagnosis adjudicated by an internal medicine specialist, applying guidelines, using all available medical records including treatment response, echo, BNP and necropsy data, when available | 53 |
| Mumoli N 2016 | LUS | 8-zones (4 anterior + 4 lateral regions). Positive LUS = ≥3 B-lines in ≥2 bilateral regions (”B-pattern”). | < 1.5 | CHF | Final hospital discharge diagnosis based on complete clinical evaluation including history, physical examination, laboratory tests, chest radiograph, ECG, echocardiography, and response to therapy. | 47 |
| Nagaharu K 2022 | LUS | 8-zones; CTS defined as multiple comet-tail artifacts in ≥1 area bilaterally | 0.03 | AHF | Diagnosis established by cardiologists when pharmacological intervention was required (diuretics, vasodilators, natriuretic peptides, and/or inotropes). Adjudication based on interview, physical assessments, BNP, chest X-ray. | 44 |
| Nakao S 2021 | LUS | 8-zones; ≥3 B-lines in ≥2 zones bilaterally defined as positive for interstitial syndrome | NA | AHF | Adjudicated by two emergency physicians using available clinical data, including labs, imaging, and response to treatment; disagreements resolved by a third reviewer | 83 |
|  | CXR | NA | NA |  |  |  |
| Neeraj G 2023 | LUS | 6-zones; BLUE protocol; upper/lower BLUE-points + PLAPS; ISS not specifically defined | 0.25 | Pulmonary oedema | Chest CT scan | 11 |
| Ocel S 2024 | LUS | BLUE protocol; thoracic US performed bilaterally at the points defined in BLUE protocol; assessed pleural sliding, A-lines, B-lines, consolidation, effusion, barcode sign | NA | Pulmonary oedema | Gold standard = CT. If CT not indicated/contraindicated, final diagnosis was determined using a combination of ultrasound, chest X-ray, clinical condition, and laboratory findings (leukocyte, CRP, BNP, etc.) | 31 |
| Oehman J 2019 | LUS | 6-zones; Bilateral scanning of apical and mammillary zones (mid-clavicular) for B-lines; basal axillary zones for pleural effusion. ≥3 B-lines in one intercostal space = positive zone. Congestion defined as bilateral B-lines or bilateral pleural fluid (>5 mm) | NA | AHF | Diagnosis required (1) structural heart disease on conventional echocardiography and (2) either BNP >400 ng/L, or BNP >100 ng/L with congestion on chest radiography evaluated by a blinded radiologist | 52 |
|  | CXR | NA | NA |  |  |  |
| Olesen AS 2022 | ReDS | >35% lung fluid content positive for congestion | < 4.8 | ADHF | Adjudicated by ≥2 cardiologists using ESC criteria (clinical signs, NT-proBNP, CXR, echo); 3rd cardiologist if disagreement | 40 |
| Panda R 2023 | CXR | NA | NA | Pulmonary oedema | Composite diagnosis made by two critical care consultants at 48 hours, after interpreting clinical and investigational data including CT scan, echocardiography, and blood investigations. | 32 |
| Patel CJ 2018 | LUS | 12-zones; BLUE protocol; patient supine/semi-recumbent; chest split into anterior, lateral, and PLAPS point; each split into upper. Pulmonary oedema (interstitial syndrome) = bilateral B+ lines in ≥2 zones with lung sliding. | NA | Pulmonary oedema | Diagnosis made by the treating unit, using chest radiography, evaluation of cardiac function by echocardiography, and clinical response to diuretics; alongside routine clinical assessment and tests (e.g., ABG, ECG; radiographs reported by radiologists) | 26 |
| Perrone T 2017 | LUS | 8-zones; bilateral anterior and lateral thorax; interstitial syndrome defined by Volpicelli criteria: ≥2 areas per hemithorax with B-lines | NA | AHF | The discharge diagnosis, as made by the attending physician, was subsequently confirmed by an independent reviewer. The attending physician was blinded to LUS results. The final diagnosis was based on clinical exam, past medical history, labs, and chest X-ray. | 72 |
|  | CXR | Reviewed by expert radiologist | NA |  |  |  |
| Pivetta E 2015 | LUS | 6-zones; Diffuse interstitial syndrome defined as bilateral presence of two or more zones with at least three B-lines. Used LUS-alone. | < 1 | ADHF | Final diagnosis adjudicated by independent review of the entire medical record by two expert emergency physicians and a cardiologist; disagreements resolved by a third expert emergency physician. | 46 |
|  | CXR | Routine, reviewed by radiologist. | NA |  |  |  |
| Prosen G 2011 | LUS | 8 zones (2 anterior + 2 lateral per side); ISS defined as ≥3 B-lines per zone; positive if ≥2 positive zones bilaterally | NA | AHF | Final diagnosis established by cardiologists and/or intensive care physicians at hospital discharge based on history, clinical examination, chest X-ray, echocardiography, cardiac functional tests, pulmonary function tests, lab tests, and invasive investigations when needed. | 60 |
| Rafique Z 2022 | ReDS | >35% lung fluid content positive for congestion | NA | Pulmonary oedema | Two emergency physicians reviewed all available data (labs, imaging, echo, notes) 30 days after enrollment to determine pulmonary oedema diagnosis. | 31 |
| Russell F 2017 | LUS | Modified LuCUS protocol = lung + cardiac US. Lung part: scan the anterosuperior lung zone in each hemithorax with a curvilinear probe; B+ lines = ≥3 B-lines in a single rib space. For this study, we use LUS-alone: positive if both left and right anterosuperior zones show B+ lines (no echo criterion applied). |  | ADHF | Final diagnosis by chart review, independently determined by two emergency physicians after reviewing labs, imaging, medications, consults, comprehensive echocardiography, and discharge summaries; disagreements resolved by a third blinded adjudicator. | 36 |
| Russell F 2023 | LUS | 4-zones (anterior–superior bilaterally), positive if ≥3 B-lines in both R1 and L1 zones; if either negative, scan zone 4 on that side. | NA | AHF | Final diagnosis based on hospital discharge diagnosis (criterion standard); no further details provided about adjudication process. | 35 |
| Saboo R 2022 | LUS | B lines pattern seen (comet tail  sign) | NA | AHF | Clinical diagnosis of heart failure based on symptoms, NT-proBNP, and imaging | 54 |
| Sartini S 2016 | LUS | 8-zones, anterior/lateral, supine/semi-recumbent. Positive if ≥3 B-lines in ≥2 zones per side. | 0.58 | AHF | Panel of two cardiologists and one emergency physician using ESC/AHA criteria incl. imaging; exact method unclear. | 48 |
|  | CXR | CXR as routine part of clinic, reviewed by on-call radiologist | 0.33 |  |  |  |
| Schoeneck JH 2021 | LUS | 8-zones BLUE protocol. Positive if B-lines bilateral (significant if more than 3 B-lines in one zone). | NA | Pulmonary oedema | Final hospital discharge diagnosis determined by treating hospital physicians after access to full diagnostic workup, including labs, imaging (CXR, CT, echo), and clinical course | 23 |
| Secko M 2022 | LUS | 12-zones, AIS = >= 3 b-lines in a given region | NA | Pulmonary oedema | Final diagnosis determined by an adjudication committee (2 EPs, 1 tiebreaker) based on full medical record: labs, echo, imaging, admission and discharge notes | 31 |
| Seibel A 2020 | LUS | 26-zones; anterior and lateral chest wall; B-lines counted in each intercostal space and averaged into a B-Line Quotient (BLQ); confluent B-lines capped at 10 per zone | NA | EVLWI | Extravascular lung water index measured by single indicator transpulmonary thermodilution (TPTD) using the PiCCO² system. > 10 mL/kg | 36 |
| Sekiguchi H 2015 | LUS | 8-zones; multiple B-line cutoffs evaluated. We report results using ≥2 zones with B-lines as threshold for ISS. | NA | Pulmonary oedema | Diagnosis based on review incl. clinical course, echocardiography, chest X-ray, CT, ABG, labs (BNP, troponin, lactate, leukocytes, creatinine), ventilation status, and vasoactive use. ARDS defined per AECC criteria. Third reviewer resolved disagreements. | 44 |
| Sen S 2017 | LUS | 12-zones; BLUE Protocol: each hemithorax divided into anterior, lateral, and posterolateral zones; each zone further subdivided into upper and lower areas (total of 6 zones per side). Scans evaluated for A-lines, B-lines, lung sliding, alveolar consolidation, and pleural effusion. | NA | CHF | Final diagnosis of CHF based on chart review by 2 board-certified physicians considering clinical judgment, radiographic, laboratory, and echocardiographic data. | 26 |
| Sforza A 2017 | LUS | 8-zones (4 zones per hemithorax: anterior and lateral); ≥3 B-lines per zone considered positive; ISS = ≥2 positive zones bilaterally + pleural effusion | NA | Cardiogenic dyspnoea | Final diagnosis of dyspnoea (cardiac vs. non-cardiac) determined by an independent emergency physician based on clinical exam, blood gas, ECG, chest X-ray, labs, and patient response to therapy; without knowledge of ultrasound findings | 40 |
|  | CXR | NA | NA |  |  |  |
| Sharma A 2025 | LUS | 8-zones (2 anterior, 2 lateral per lung); ≥2 bilateral B-lines = positive (Cardinale et al.) | NA | AHF | Final hospital diagnosis based on clinical criteria, chest X-ray, echocardiography, cardiac tests | 43 |
| Siddiqi M 2025 | LUS | Posterior lung scanning only; patient in left lateral or sitting position; probe moved across lung fields bilaterally; diagnostic criteria included B-lines for oedema, liver-like echogenicity for pneumonia, anechoic space for effusion, etc. | NA | Pulmonary oedema | Final clinical diagnosis after extensive workup and clinical examination by emergency specialists. | 31 |
| Studler U 2008 | CXR | Erect position, the radiologist’s overall impression of heart failure. | NA | AHF | Final discharge diagnosis adjudicated by an internal medicine specialist using all available medical records, including echocardiography, BNP. | 56 |
| Tækker M 2022 | CXR | Supine chest X-ray | < 4 | Pulmonary oedema | Non-contrast chest CT interpreted by chest radiologist with 10 years of experience | 10 |
| Tsverava M 2010 | LUS | 3 and more comet tails | NA | Acute diastolic heart failure | DHF defined by EF > 50% plus Echo-Doppler evidence of LV diastolic dysfunction (sinus rhythm) or EF > 50% in AF with EchoDoppler signs; LV diastolic function categorised by transmitral/pulmonary venous/TDI/Color M-mode. | 23 |
| Uriel N 2018 | ReDS | Optimal cut-off 34% lung fluid content | NA | PAWP | Invasively measured pulmonary artery wedge pressure during right heart catheterisation, positive >18 mmHg | 31 |
| Vauthier C 2021 | LUS | 12-zones; B-profile defined as ≥3 B-lines in ≥2 zones on each lung |  | AHF | Final diagnosis adjudicated by 2 emergency physicians based on full clinical record (including natriuretic peptides, echocardiography, response to treatment), blinded to LUS results. | 41 |
| Vitturi N 2011 | LUS | Frontal and longitudinal scans along parasternal, mid-clavicular, and mid-axillary lines (supine or semi-sitting). Positive if >8 B-lines (comet-tail artifacts) detected. Each exam <3 min. | NA | AHF | Final clinical diagnosis of heart failure at hospital discharge (based on all available clinical, imaging, laboratory, and echocardiographic data) | 45 |
|  | CXR | NA | NA |  |  |  |
| Volpicelli G 2014 | LUS | 8-zones; anterior-lateral, 2–5 MHz curvilinear probe. B-pattern defined as ≥3 B-lines in ≥2 zones per side considered positive. A-pattern (normal aeration or focal findings) considered negative. |  | EVLWI | Extravascular lung water index (EVLWI) measured via transpulmonary thermodilution (PiCCO system); EVLWI >10 mL/kg considered abnormal | 66 |
| Wooten WM 2018 | LUS | 8-zones; anterior/lateral bilaterally. Positive if ≥3 B-lines in ≥2 zones per side. | 1.37 | Pulmonary oedema | Discharge summaries that included: “pulmonary oedema,” “fluid overload,” “congestive heart failure,” “chronic kidney disease,” and “liver disease” | 52 |
|  | CXR | NA | NA |  |  |  |
| Yang F 2017 | LUS | 28-zone scanning; sum of B-lines used as score (cut-off ≥30 for HFrEF to predict E/e′ ≥14) | NA | E/e’ | Ratio of early transmitral flow velocity (E) to early diastolic mitral annular velocity (e′), measured by echocardiography. An E/e′ ≥ 14 was used as the diagnostic cut-off for elevated left ventricular filling pressure according to 2016 ESC heart failure guidelines | NA |
| Zhong L 2012 | LUS | 8 zones per lung (Volpicelli method); ≥3 B-lines in ≥2 zones on one side = positive | < 5 | AHF | Diagnosis of acute cardiac or pulmonary dyspnoea made by a clinical physician, based on medical history, physical exam, blood tests, chest X-ray, CT, echocardiography, and pulmonary function tests. | 55 |
| Zhou S 2014 | LUS | 12 zones (6 per side); A-line = horizontal lines indicating normal lung; B-line = vertical lines from pleura indicating interstitial syndrome; ≥3 B-lines in a zone = positive region | < 24 | Pulmonary oedema | Final diagnosis based on clinical assessment, imaging (X-ray, sometimes CT), and/or expert consultation. Used to determine if pulmonary oedema was present. | 35 |
|  | CXR | NA | < 24 |  |  |  |
| Zhu L 2024 | LUS | 28 zones; supine position. B-lines counted (single, B7, B3, white lung). Pleural effusion scored (moderate/massive). Max score = 30 | NA | Pulmonary oedema | Diagnosis of heart failure with pulmonary oedema according to the 2021 ESC Guidelines for the diagnosis and management of heart failure, including NYHA functional classification | 81 |

ACS: Acute Coronary Syndrome, ADHF: Acute Decompensated Heart Failure, AECOPD: Acute Exacerbation of Chronic Obstructive Pulmonary Disease, AF: Atrial Fibrillation, AIS: Alveolar-Interstitial Syndrome, APE: Acute Pulmonary Oedema, ASE: American Society of Echocardiography, BBPC: Bedside BLUE-Plus-Cardiac method, BNP: B-type Natriuretic Peptide, BLUE: Bedside Lung Ultrasound in Emergency protocol, CHF: Congestive Heart Failure, COPD: Chronic Obstructive Pulmonary Disease, CT: Computed Tomography, CVC: Central Venous Catheter, DHF: Diastolic Heart Failure, ECG: Electrocardiogram, ED: Emergency Department, EF: Ejection Fraction, EM: Emergency Medicine, EP: Emergency Physician, ESC: European Society of Cardiology, EVLW: Extravascular Lung Water, EVLWI: Extravascular Lung Water Index, HF: Heart Failure, HFrEF: Heart Failure with Reduced Ejection Fraction, ICS: Intercostal Space, ISS: Interstitial Syndrome Score, LDCT: Low-Dose Computed Tomography, LHC: Left Heart Catheterisation, LUS: Lung Ultrasound, LV: Left Ventricle, LVEDP: Left Ventricular End-Diastolic Pressure, LVEF: Left Ventricular Ejection Fraction, NA: Not Available / Not Applicable, PAOH: Pulmonary Artery Occlusion Hypertension, PAOP: Pulmonary Artery Occlusion Pressure, PAWP: Pulmonary Artery Wedge Pressure, PCWP: Pulmonary Capillary Wedge Pressure, PiCCO: Pulse Contour Cardiac Output monitor, ReDS: Remote Dielectric Sensing, TDI: Tissue Doppler Imaging, TPTD: Transpulmonary Thermodilution, ULC: Ultrasound Lung Comets

| **Supplemental** **Table 3.** Comorbidities and Symptoms | | | | | | | | | | | | | | | | | |
| --- | --- | --- | --- | --- | --- | --- | --- | --- | --- | --- | --- | --- | --- | --- | --- | --- | --- |
| Study | HTN, % | DM, % | COPD, % | AFLI, % | HF, % | CKD, % | IHD, % | NYHA II, % | NYHA III, % | NYHA IV, % | Orthopnoea, % | Paroxysmal nocturnal dyspnoea, % | Peripheral oedema, % | Cough, % | Fever, % | Rales, % | Wheezing, % |
| Aggarwal M 2016 | - | - | - | - | - | - | - | - | - | - | - | - | - | - | - | - | - |
| Ali M 2022 | 38 | . | 20 | - | 13 | 6 | 16 | - | - | - | - | - | - | - | - | - | - |
| Anderson KL 2013 | - | 33 | 29 | - | 51 | 19 | 28 | - | - | - | 50 | 19 | 48 | - | - | 36 | 25 |
| Arthur M 2022 | 29 | 34 | 7 | - | - | - | - | - | - | - | - | - | - | 34 | 57 | - | - |
| Azab NY 2022 | - | - | - | - | - | - | - | - | - | - | - | - | - | - | - | - | - |
| Baid H 2022 | 31 | 27 | 6 | - | - | 6 | 6 | - | - | - | - | - | - | - | - | - | - |
| Baker K 2016 | - | - | - | - | - | - | - | - | - | - | - | - | - | - | - | - | - |
| Baldi G 2013 | - | - | - | - | - | - | - | - | - | - | - | - | - | - | - | - | - |
| Balen F 2021 | - | - | 17 | 33 | 63 | 6 | - | - | - | - | - | - | - | - | 22 | - | - |
| Bataille B 2014 | - | - | - | - | - | - | - | - | - | - | - | - | - | - | - | - | - |
| Bekgoz 2019 | 23 | 16 | 23 | - | 15 | - | 19 | - | - | - | - | - | - | - | - | - | - |
| Bitar Z 2015 | 84 | 85 | 16 | 16 | - | 39 | 37 | - | - | - | - | - | - | - | - | 54 | 30 |
| Bouzidi H 2025 | 56 | 41 | 15 | - | 44 | 28 | 23 | - | - | - | - | - | - | - | - | - | - |
| Buessler A 2019 | - | - | 33 | - | 16 | - | - | - | 44 | 50 | - | - | 54 | - | - | 44 | 7 |
| Buhumaid RE 2019 | - | - | 26 | - | 23 | - | 34 | - | - | - | - | - | - | - | - | - | - |
| Carlino MV 2018 | - | - | 33 | - | - | - | - | - | - | - | - | - | - | - | - | - | - |
| Chaitra S 2022 | - | - | - | - | - | - | - | - | - | - | - | - | - | 8 | 25 | - | - |
| Chiem E 2015 | - | - | - | - | 46 | - | - | - | - | - | - | - | 42 | - | - | 34 | - |
| Cibinel GA 2012 | - | - | 39 | - | 48 | - | - | - | - | - | - | - | - | - | - | - | - |
| Coiro S 2016 | - | - | - | - | - | - | 46 | - | - | - | - | - | - | - | - | - | - |
| De Carvalho H 2021 | 70 | 13 | 35 | - | 64 | - | - | - | - | - | - | - | - | - | - | - | - |
| Dehbozorgi A 2019 | 27 | 37 | 18 | - | 9 | - | 36 | - | - | - | - | - | - | - | 18 | - | - |
| Dexheimer N 2015 | - | - | 22 | - | 19 | 11 | - | - | - | - | - | - | - | - | - | - | - |
| Elatroush HH 2023 | 51 | 58 | 10 | 5 | - | 11 | 15 | - | - | - | - | - | - | - | - | - | - |
| Elmahalawy II 2017 | 28 | 24 | - | 2 | - | 23 | - | - | - | - | - | - | - | - | - | - | - |
| Elnaem WH 2017 | - | - | - | - | - | - | - | - | - | - | - | - | - | - | - | - | - |
| Ely W 2001 | - | - | - | - | - | - | - | - | - | - | - | - | - | - | - | - | - |
| Farahmand S 2019 | - | 4 | 7 | - | 17 | 3 | 11 | - | - | - | 63 | 47 | 42 | - | - | 42 | 14 |
| Feuerstein D 2022 | 46 | 18 | 14 | 20 | 8 | 18 | 30 | - | - | - | - | - | - | - | - | - | - |
| Gallard E 2015 | - | - | - | - | - | - | - | - | - | - | - | - | - | - | - | - | - |
| Gargani L 2008 | 53 | 37 | 22 | - | 31 | 29 | 40 | 43 | 37 | 20 | - | - | 32 | - | - | 29 | - |
| Ghauri S 2021 | - | - | 12 | - | 46 | - | - | - | - | - | - | - | - | - | - | - | - |
| Gillespie ND 1997 | 25 | - | - | - | - | - | 32 | - | - | - | - | - | - | - | - | - | - |
| Glöckner E 2020 | 80 | 33 | 28 | - | 70 | 37 | - | - | - | - | - | - | - | - | - | - | - |
| Heldeweg MLA 2022 | - | - | - | - | - | - | - | - | - | - | - | - | - | - | - | - | - |
| Hubert A 2019 | 69 | 21 | 7 | 31 | 51 | 30 | - | 73 | - | - | - | - | - | - | - | - | - |
| Imamura T 2022 | 73 | 27 | - | 40 | 100 | 60 | 7 | - | - | - | - | - | - | - | - | - | - |
| Inui G 2024 | - | - | - | - | - | - | - | - | - | - | - | - | - | 9 | 14 | - | 4 |
| Izumida T 2022 | 80 | 40 | 9 | 45 | 100 | 78 | 22 | - | - | - | - | - | - | - | - | - | - |
| Izumida T 2023 | 53 | 32 | - | 42 | 100 | - | 35 | 68 | 16 | 0 | - | - | - | - | - | - | - |
| Johannessen Ø 2023 | 36 | 13 | 100 | - | 13 | - | 27 | - | - | - | - | - | - | - | - | - | - |
| Joseph RB 2024 | - | - | - | - | - | - | - | - | - | - | - | - | - | - | - | - | - |
| Kennedy S 2011 | - | - | - | - | - | - | - | - | - | - | - | - | - | - | - | - | - |
| Knudsen CW 2004 | 62 | - | - | - | 38 | - | 28 | - | - | - | 55 | - | 45 | - | - | 41 | - |
| Koh Y 2018 | - | - | - | - | - | - | - | - | - | - | 27 | 27 | - | 68 | 26 | - | - |
| Lajili F 2024 | 45 | 35 | - | - | 18 | 8 | 13 | 23 | 41 | 20 | 20 | - | - | - | 17 | - | - |
| Laursen CB 2016 | 48 | 20 | 53 | - | 10 | - | 33 | - | - | - | - | - | - | - | - | - | - |
| Lichtensten DA 2008 | - | - | - | - | - | - | - | - | - | - | - | - | - | - | - | - | - |
| Lichtensten DA 2009 | - | - | - | - | - | - | - | - | - | - | - | - | - | - | - | - | - |
| Liteplo AS 2009 | 40 | 22 | 31 | 26 | 49 | 18 | - | - | - | - | - | - | - | - | - | - | - |
| Liu ZP 2016 | - | - | - | - | - | - | - | - | - | - | - | - | - | - | - | - | - |
| Martinez-Rumayor AA 2010 | 49 | 26 | 12 | - | 25 | - | - | - | - | - | 17 | 12 | 18 | 37 | 9 | 26 | 25 |
| Mayr U 2022 | - | - | - | - | - | - | - | - | - | - | - | - | - | - | - | - | - |
| Miger K 2019 | 62 | 24 | 52 | 36 | 27 | 7 | 27 | 28 | 45 | 21 | 50 | - | 33 | 84 | 10 | 41 | - |
| Miger K 2024 | - | - | - | - | 25 | - | - | 32 | 45 | 23 | 49 | 47 | 31 | - | - | 39 | - |
| Modi UP 2024 | - | - | - | - | - | - | - | - | - | - | - | - | - | - | - | - | - |
| Msolli MA 2021 | 49 | 41 | 22 | - | 25 | - | 19 | - | - | - | - | - | - | - | - | - | - |
| Mueller-Lenke N 2006 | - | - | - | - | - | - | - | - | - | - | - | - | - | - | - | - | - |
| Mumoli N 2016 | - | - | - | - | - | - | - | - | - | - | - | - | - | - | - | - | - |
| Nagaharu K 2022 | - | - | - | - | - | - | - | - | - | - | - | - | 46 | - | - | - | - |
| Nakao S 2021 | 75 | 43 | 14 | 32 | 36 | 26 | 37 | - | - | - | - | - | - | - | - | - | - |
| Neeraj G 2023 | 19 | 9 | - | - | 10 | 16 | - | - | - | - | - | - | - | 11 | 11 | - | - |
| Ocel S 2024 | - | - | - | - | - | - | - | - | - | - | - | - | - | - | - | - | - |
| Öhman J 2019 | 70 | 34 | 34 | - | 41 | 30 | 39 | - | - | - | - | - | - | - | - | 28 | 22 |
| Olesen ASO 2022 | - | 26 | 53 | - | 26 | 11 | - | 31 | 44 | 25 | 50 | 61 | 28 | - | 16 | 20 | 12 |
| Panda R 2023 | - | - | - | - | - | - | - | - | - | - | - | - | - | - | - | - | - |
| Patel CJ 2018 | - | - | - | - | - | - | - | - | - | - | - | - | - | - | - | - | - |
| Perrone T 2017 | - | - | - | - | - | - | - | 38 | 36 | 19 | - | - | 34 | - | 30 | 77 | - |
| Pivetta E 2015 | 64 | 28 | 41 | - | 20 | 17 | 26 | - | - | - | - | - | 37 | 35 | 19 | 66 | 31 |
| Prosen G 2011 | - | - | - | - | - | - | - | - | - | - | - | - | - | - | - | - | - |
| Rafique Z 2022 | 48 | 25 | 19 | - | 29 | 10 | - | - | - | - | - | - | - | - | - | - | - |
| Russell F 2017 | 69 | 29 | 43 | - | 40 | - | 16 | - | - | - | - | - | - | - | - | - | - |
| Russell F 2023 | 69 | - | 57 | - | 49 | 24 | 31 | - | - | - | - | - | - | - | - | - | - |
| Saboo R 2022 | - | - | - | - | - | - | - | - | - | - | - | - | - | - | - | - | - |
| Sartini S 2016 | - | - | - | - | - | - | - | - | - | - | - | - | - | - | - | - | - |
| Schoeneck JH 2021 | - | - | - | - | - | - | - | - | - | - | - | - | - | - | - | - | - |
| Secko M 2022 | 52 | 22 | 29 | - | - | 10 | 22 | - | - | - | - | - | - | - | - | - | - |
| Seibel A 2020 | - | - | 18 | - | 17 | - | - | - | - | - | - | - | - | - | - | - | - |
| Sekiguchi H 2015 | - | - | - | - | - | - | - | - | - | - | - | - | - | - | - | - | - |
| Sen S 2017 | - | - | 32 | - | 16 | - | - | - | - | - | - | - | - | - | - | - | - |
| Sforza A 2017 | - | - | 49 | 41 | - | - | - | - | - | - | - | - | - | - | - | - | - |
| Sharma A 2025 | 39 | 39 | 41 | - | 23 | - | 16 | - | - | - | 48 | 38 | 45 | 83 | 50 | 69 | 52 |
| Siddiqi M 2025 | 25 | 17 | - | - | - | 11 | - | - | - | - | - | - | - | - | - | - | - |
| Studler U 2008 | 53 | - | 31 | - | - | - | 50 | - | - | - | - | - | - | 53 | 24 | - | - |
| Tsverava M 2010 | - | - | - | - | - | - | - | 20 | 52 | 28 | 42 | 35 | - | - | - | 42 | 2 |
| Tækker M 2022 | - | - | - | - | - | - | - | - | - | - | - | - | - | - | - | - | - |
| Uriel 2018 | 63 | 36 | 6 | 27 | - | - | - | - | - | - | - | - | - | - | - | - | - |
| Vauthier C 2021 | - | - | 21 | - | 26 | - | - | - | - | - | - | - | - | - | - | - | - |
| Vitturi N 2011 | - | - | - | - | - | - | - | - | - | - | - | - | - | - | - | - | - |
| Volpicelli G 2014 | - | - | - | - | - | - | - | - | - | - | - | - | - | - | - | - | - |
| Wooten WM 2018 | 79 | 41 | 40 | 25 | 32 | 24 | 31 | - | - | - | - | - | - | - | - | - | - |
| Yang F 2017 | 76 | 56 | 4 | - | 100 | - | 62 | - | 61 | 39 | - | - | - | - | - | - | - |
| Zhong L 2012 | - | - | 91 | - | 41 | - | - | - | - | - | - | - | - | - | - | - | - |
| Zhou S 2014 | - | - | - | - | - | - | - | - | - | - | - | - | - | - | - | - | - |
| Zhu L 2024 | - | - | - | - | - | - | - | 34 | 36 | 4 | - | - | - | - | - | - | - |

HTN: Hypertension, DM: Diabetes Mellitus, COPD: Chronic Obstructive Pulmonary Disease, AFLI: Atrial Fibrillation, HF: Heart Failure, CKD: Chronic Kidney Disease, IHD: Ischemic Heart Disease, NYHA: New York Heart Association

| **Supplemental Table 4.** Admission variables | | | | | | | | | | |
| --- | --- | --- | --- | --- | --- | --- | --- | --- | --- | --- |
| Study | SBP, mmHg | DBP, mmHg | Heart rate, /min | Respiratory rate, /min | Oxygen saturation, % | Temperature, °C | LVEF, mean % | LVEF, median % | NT-proBNP | Unit |
| Aggarwal M 2016 | - | - | - | - | - | - | - | - | - | - |
| Ali M 2022 | - | - | - | - | - | - | - | - | - | - |
| Anderson KL 2013 | - | - | - | - | - | - | - | - | - | - |
| Arthur M 2022 | - | - | - | - | - | - | - | - | - | - |
| Azab NY 2022 | - | - | - | - | - | - | - | - | - | - |
| Baid H 2022 | 127 | 79 | 106 | 26 | 83 | 37.3 | - | - | - | - |
| Baker K 2016 | - | - | - | - | - | - | - | - | - | - |
| Baldi G 2013 | - | - | - | - | - | - | - | - | - | - |
| Balen F 2021 | 145 | - | 92 | 28 | 85 | - | - | - | 3359 | pg/mL |
| Bataille B 2014 | - | - | - | - | - | - | - | - | - | - |
| Bekgoz 2019 | - | - | - | - | - | - | - | - | - | - |
| Bitar Z 2015 | - | - | - | - | - | - | - | - | - | - |
| Bouzidi H 2025 | - | - | - | - | - | - | 44 | - | 14125 | pg/mL |
| Buessler A 2019 | 137 | 73 | 94 | 27 | 94 | - | - | - | 2815 | pg/mL |
| Buhumaid RE 2019 | - | - | - | - | - | - | - | - | - | - |
| Carlino MV 2018 | 137 | 75 | 90 | 27 | 90 | - | 51 | - | 5551 | pg/mL |
| Chaitra S 2022 | - | - | - | - | - | - | - | - | - | - |
| Chiem E 2015 | - | - | - | - | - | - | - | - | - | - |
| Cibinel GA 2012 | - | - | - | - | - | - | - | - | - | - |
| Coiro S 2016 | - | - | - | - | - | - | 39 | - | BNP 709 | pg/mL |
| De Carvalho H 2021 | 141 | 78 | 100 | 29 | 91 | 37 | - | - | - | - |
| Dehbozorgi A 2019 | - | - | - | - | - | - | - | - | - | - |
| Dexheimer N 2015 | - | - | - | - | - | - | - | - | - | - |
| Elatroush HH 2023 | - | - | - | - | - | - | - | - | - | - |
| Elmahalawy II 2017 | - | - | - | - | - | - | - | - | - | - |
| El-em WH 2017 | - | - | - | - | - | - | - | - | - | - |
| Ely W 2001 | - | - | - | - | - | - | - | - | - | - |
| Farahmand S 2019 | - | - | - | - | - | - | - | - | - | - |
| Feuerstein D 2022 | - | - | - | - | - | - | - | - | - | - |
| Gallard E 2015 | 150 | 82 | 90 | 24 | 93 | 37.3 | - | - | - | - |
| Gargani L 2008 | 131 | 73 | 78 | - | - | - | - | - | - | - |
| Ghauri S 2021 | - | - | - | - | - | - | - | - | - | - |
| Gillespie ND 1997 | - | - | - | - | - | - | - | - | - | - |
| Glöckner E 2020 | 136 | - | 86 | 17 | 96 | - | - | - | 2648 | pg/mL |
| Heldeweg MLA 2022 | - | - | - | - | - | - | - | - | BNP 1771 | - |
| Hubert A 2019 | 135 | 74 | 73 | - | - | - | - | 52 | 2014 | pg/mL |
| Imamura T 2022 | - | - | 70 | - | - | - | - | 54 | BNP 166 | pg/mL |
| Inui G 2024 | - | - | - | - | - | - | - | - | - | - |
| Izumida T 2022 | - | - | - | - | - | - | - | 45 | BNP 205 | pg/mL |
| Izumida T 2023 | - | - | - | - | - | - | - | 50 | BNP 131 | pg/mL |
| Johannessen Ø 2023 | 139 | - | - | 27 | 89 | - | - | - | 455 | ng/L |
| Joseph RB 2024 | - | - | - | - | - | - | - | - | - | - |
| Kennedy S 2011 | - | - | - | - | - | - | - | - | - | - |
| Knudsen CW 2004 | - | - | - | - | - | - | - | - | - | - |
| Koh Y 2018 | - | - | - | - | - | - | - | - | - | - |
| Lajili F 2024 | 138 | 75 | 104 | 26 | 86 | - | - | - | BNP 161 | pg/mL |
| Laursen CB 2016 | - | - | - | - | - | - | - | - | - | - |
| Lichtensten DA 2008 | - | - | - | - | - | - | - | - | - | - |
| Lichtensten DA 2009 | - | - | - | - | - | - | - | - | - | - |
| Liteplo AS 2009 | - | - | - | - | - | - | - | - | - | - |
| Liu ZP 2016 | 130 | 76 | 101 | - | - | - | 47 | - | - | - |
| Martinez-Rumayor AA 2010 | - | - | - | - | - | - | - | - | - | - |
| Mayr U 2022 | - | - | - | - | - | - | - | - | - | - |
| Miger K 2019 | 146 | 78 | 93 | 22 | - | - | 50 | - | 3624 | ng/L |
| Miger K 2024 | 144 | - | - | - | - | - | 51 | - | 1356 | pg/ml |
| Modi UP 2024 | - | - | - | - | - | - | - | - | - | - |
| Msolli MA 2021 | - | - | - | - | - | - | 51 | - | BNP 216 | pg/ml |
| Mueller-Lenke N 2006 | - | - | - | - | - | - | - | - | - | - |
| Mumoli N 2016 | - | - | - | - | - | - | - | - | - | - |
| Nagaharu K 2022 | - | - | - | - | - | - | - | - | BNP 271 | pg/mL |
| Nakao S 2021 | 140 | - | 89 | 24 | 92 | 36.2 | - | - | NA | NA |
| Neeraj G 2023 | - | - | - | - | - | - | - | - | - | - |
| Ocel S 2024 | - | - | - | - | - | - | - | - | - | - |
| Öhman J 2019 | 140 | - | 86 | 23 | - | - | 46 | - | BNP 367 | ng/L |
| Olesen ASO 2022 | - | - | 90 | - | - | - | - | 55 | 998 | ng/L |
| Panda R 2023 | - | - | - | - | - | - | - | - | - | - |
| Patel CJ 2018 | - | - | - | - | - | - | - | - | - | - |
| Perrone T 2017 | - | - | 98 | 28 | 92 | - | 45 | - | - | - |
| Pivetta E 2015 | 143 | 79 | 97 | 28 | - | 36.6 | - | - | - | - |
| Prosen G 2011 | - | - | - | - | - | - | - | - | 1583 | pg/mL |
| Rafique Z 2022 | 136 | 82 | - | - | 98 | - | - | - | - | - |
| Russell F 2017 | - | - | - | - | - | - | - | - | - | - |
| Russell F 2023 | 152 | - | 101 | 23 | 91 | - | - | - | - | - |
| Saboo R 2022 | - | - | - | - | 77 | - | - | - | 6399 | pg/mL |
| Sartini S 2016 | 132 | 73 | 90 | 21 | 92 | - | - | NA | 5552 | pg/ml |
| Schoeneck JH 2021 | - | - | 93 | 23 | 92 | - | - | - | - | - |
| Secko M 2022 | - | - | - | - | - | - | - | - | - | - |
| Seibel A 2020 | - | - | - | - | - | - | - | - | - | - |
| Sekiguchi H 2015 | - | - | - | - | - | - | - | - | 3398 | pg/mL |
| Sen S 2017 | - | - | - | - | - | - | - | - | - | - |
| Sforza A 2017 | 135 | 78 | 97 | 24 | 92 | 37 | - | - | - | - |
| Sharma A 2025 | - | - | - | - | - | - | - | - | 457 | pg/mL |
| Siddiqi M 2025 | 132 | - | 96 | 23 | 91 | 38 | - | - | - | - |
| Studler U 2008 | - | - | - | - | - | - | - | - | BNP 537 | pg/mL |
| Tsverava M 2010 | 149 | 87 | 85 | - | - | - | 40 | - | - | NA |
| Tækker M 2022 | - | - | - | - | - | - | - | - | - | - |
| Uriel 2018 | - | - | - | - | - | - | - | - | - | - |
| Vauthier C 2021 | 136 | - | 95 | 26 | 91 | 37 | - | - | - | - |
| Vitturi N 2011 | - | - | - | - | 90 | - | - | - | - | - |
| Volpicelli G 2014 | - | - | - | - | - | - | - | - | - | - |
| Wooten WM 2018 | - | - | - | - | - | - | - | - | - | - |
| Yang F 2017 | 141 | 67 | 80 | - | - | - | 44 | - | 8928 | pg/mL |
| Zhong L 2012 | - | - | 85 | - | - | - | - | - | BNP 1095 | pg/mL |
| Zhou S 2014 | - | - | - | - | - | - | - | - | - | - |
| Zhu L 2024 | - | - | - | - | - | - | - | - | - | - |

| **Supplemental Table 5.** Admission Causes | | | | |
| --- | --- | --- | --- | --- |
| Study | Acute heart failure, % | COPD exacerbation, % | Pneumonia, % | Other, % |
| Aggarwal M 2016 | 81 | - | - | 19 |
| Ali M 2022 | 24 | - | 19 | 57 |
| Anderson KL 2013 | 44 | 8 | 10 | 38 |
| Arthur M 2022 | - | 3 | 30 | - |
| Azab NY 2022 | 12 | 14 | 44 | 30 |
| Baid H 2022 | 15 | 7 | 79 | 0 |
| Baker K 2016 | 20 | 30 | 19 | 31 |
| Baldi G 2013 | - | - | 25 | - |
| Balen F 2021 | 65 | 6 | 20 | 31 |
| Bataille B 2014 | 25 | 3 | 57 | 15 |
| Bekgoz 2019 | 25 | 28 | 24 | 23 |
| Bitar Z 2015 | 74 | - | - | 26 |
| Bouzidi H 2025 | 79 | - | - | 21 |
| Buessler A 2019 | 62 | - | - | 38 |
| Buhumaid RE 2019 | 15 | 16 | 11 | 58 |
| Carlino MV 2018 | 39 | 20 | 15 | 26 |
| Chaitra S 2022 | 22 | 13 | 32 | 33 |
| Chiem E 2015 | 35 | - | - | 65 |
| Cibinel GA 2012 | 48 | - | - | 52 |
| Coiro S 2016 | 100 | - | - | 0 |
| De Carvalho H 2021 | 48 | 8 | 27 | 17 |
| Dehbozorgi A 2019 | 28 | 27 | - | 45 |
| Dexheimer N 2015 | 41 | 11 | 46 | 2 |
| Elatroush HH 2023 | 4 | - | - | 96 |
| Elmahalawy II 2017 | 15 | - | 68 | - |
| Elnaem WH 2017 | 33 | - | - | - |
| Ely W 2001 | 66 | - | - | 34 |
| Farahmand S 2019 | 48 | - | - | 52 |
| Feuerstein D 2022 | 24 | 14 | 4 | 58 |
| Gallard E 2015 | 62 | - | - | 38 |
| Gargani L 2008 | 82 | 7 | - | 6 |
| Ghauri S 2021 | 55 | - | - | 45 |
| Gillespie ND 1997 | 63 | - | - | 37 |
| Glöckner E 2020 | 54 | - | - | 46 |
| Heldeweg MLA 2022 | 27 | - | - | 73 |
| Hubert A 2019 | 100 | - | - | 0 |
| Imamura T 2022 | 100 | - | - | 0 |
| Inui G 2024 | 74 | - | 26 | 0 |
| Izumida T 2022 | 100 | - | - | 0 |
| Izumida T 2023 | 100 | 0 | 0 | 0 |
| Johannessen Ø 2023 | 38 | 100 | - | - |
| Joseph RB 2024 | 19 | - | 35 | 46 |
| Kennedy S 2011 | 44 | - | - | 56 |
| Knudsen CW 2004 | 51 | - | - | 49 |
| Koh Y 2018 | 33 | 8 | 21 | 38 |
| Lajili F 2024 | 41 | - | - | 59 |
| Laursen CB 2016 | 45 | - | - | 55 |
| Lichtensten DA 2008 | 25 | 19 | 32 | 24 |
| Lichtensten DA 2009 | 13 | - | - | 87 |
| Liteplo AS 2009 | 21 | 10 | 11 | 58 |
| Liu ZP 2016 | 47 | 0 | 100 | 0 |
| Martinez-Rumayor AA 2010 | 35 | - | - | 65 |
| Mayr U 2022 | - | - | 34 | - |
| Miger K 2019 | 27 | - | - | 73 |
| Miger K 2024 | 28 | - | - | - |
| Modi UP 2024 | 30 | 15 | 40 | 15 |
| Msolli MA 2021 | 53 | 21 | 8 | 18 |
| Mueller-Lenke N 2006 | 53 |  |  | 47 |
| Mumoli N 2016 | 47 | 21 | 22 | 10 |
| Nagaharu K 2022 | 44 | 5 | 17 | 34 |
| Nakao S 2021 | - | - | - | 3 |
| Neeraj G 2023 | 11 | 11 | 13 | 65 |
| Ocel S 2024 | 31 | - | 60 | 9 |
| Öhman J 2019 | 52 | - | - | 48 |
| Olesen ASO 2022 | 40 | - | - | 60 |
| Panda R 2023 | 32 | - | - | 68 |
| Patel CJ 2018 | 26 | 28 | 34 | 12 |
| Perrone T 2017 | 62 | - | - | - |
| Pivetta E 2015 | 46 | - | - | 54 |
| Prosen G 2011 | 60 | - | - | 40 |
| Rafique Z 2022 | 15 | 7 | 1 | 77 |
| Russell F 2017 | 36 | 24 | 10 | 30 |
| Russell F 2023 | 35 | 32 | 13 | 20 |
| Saboo R 2022 | 54 | 30 | 2 | 14 |
| Sartini S 2016 | 48 | 17 | 11 | 24 |
| Schoeneck JH 2021 | 23 | 32 | 8 | 37 |
| Secko M 2022 | 31 | 28 | 20 | 21 |
| Seibel A 2020 | - | - | - | - |
| Sekiguchi H 2015 | 44 | 3 | 31 | 22 |
| Sen S 2017 | 26 | 6 | 54 | 14 |
| Sforza A 2017 | 37 | 25 | 27 | 11 |
| Sharma A 2025 | 43 | - | - | 57 |
| Siddiqi M 2025 | 31 | - | 23 | 46 |
| Studler U 2008 | 56 | 15 | - | 29 |
| Tsverava M 2010 | - | - | - | - |
| Tækker M 2022 | 10 | - | 11 | 79 |
| Uriel 2018 | - | - | - | - |
| Vauthier C 2021 | 41 | 16 | 24 | 19 |
| Vitturi N 2011 | 45 | - | - | 55 |
| Volpicelli G 2014 | 14 | 14 | 7 | 65 |
| Wooten WM 2018 | 52 | - | - | 48 |
| Yang F 2017 | 100 | - | - | 0 |
| Zhong L 2012 | 55 | 31 | - | 14 |
| Zhou S 2014 | 35 | 36 | 14 | 15 |
| Zhu L 2024 | 81 | - | - | 19 |

## **Supplemental Table 6.** Sensitivity analyses of prior specifications for the Bayesian analysis

| **Model** | **Index test** | **Sens (mean)** | **Spec (mean)** | **SD sens** | **SD sens 2.5%** | **SD sens 97.5%** | **SD spec** | **SD spec 2.5%** | **SD spec 97.5%** | **Corr**  **sens,spec** | **Corr 2.5%** | **Corr 97.5%** |
| --- | --- | --- | --- | --- | --- | --- | --- | --- | --- | --- | --- | --- |
| Baseline | CT | 0.765 | 0.937 | 1.27 | 1.06 | 1.53 | 1.10 | 0.92 | 1.32 | -0.09 | -0.31 | 0.14 |
| SD tighter | CT | 0.768 | 0.938 | 1.26 | 1.05 | 1.51 | 1.09 | 0.91 | 1.30 | -0.09 | -0.32 | 0.14 |
| SD looser | CT | 0.759 | 0.938 | 1.28 | 1.07 | 1.54 | 1.11 | 0.93 | 1.33 | -0.09 | -0.32 | 0.14 |
| FE tighter | CT | 0.753 | 0.927 | 1.27 | 1.06 | 1.53 | 1.10 | 0.92 | 1.32 | -0.09 | -0.31 | 0.15 |
| FE looser | CT | 0.764 | 0.939 | 1.27 | 1.07 | 1.53 | 1.10 | 0.92 | 1.31 | -0.09 | -0.31 | 0.13 |
| LKJ4 | CT | 0.762 | 0.938 | 1.27 | 1.06 | 1.52 | 1.10 | 0.92 | 1.31 | -0.08 | -0.31 | 0.13 |
| Baseline | CXR | 0.622 | 0.852 | 1.27 | 1.06 | 1.53 | 1.10 | 0.92 | 1.32 | -0.09 | -0.31 | 0.14 |
| SD tighter | CXR | 0.624 | 0.854 | 1.26 | 1.05 | 1.51 | 1.09 | 0.91 | 1.30 | -0.09 | -0.32 | 0.14 |
| SD looser | CXR | 0.617 | 0.855 | 1.28 | 1.07 | 1.54 | 1.11 | 0.93 | 1.33 | -0.09 | -0.32 | 0.14 |
| FE tighter | CXR | 0.618 | 0.850 | 1.27 | 1.06 | 1.53 | 1.10 | 0.92 | 1.32 | -0.09 | -0.31 | 0.15 |
| FE looser | CXR | 0.621 | 0.855 | 1.27 | 1.07 | 1.53 | 1.10 | 0.92 | 1.31 | -0.09 | -0.31 | 0.13 |
| LKJ4 | CXR | 0.618 | 0.855 | 1.27 | 1.06 | 1.52 | 1.10 | 0.92 | 1.31 | -0.08 | -0.31 | 0.13 |
| Baseline | LUS | 0.857 | 0.894 | 1.27 | 1.06 | 1.53 | 1.10 | 0.92 | 1.32 | -0.09 | -0.31 | 0.14 |
| SD tighter | LUS | 0.858 | 0.896 | 1.26 | 1.05 | 1.51 | 1.09 | 0.91 | 1.30 | -0.09 | -0.32 | 0.14 |
| SD looser | LUS | 0.855 | 0.896 | 1.28 | 1.07 | 1.54 | 1.11 | 0.93 | 1.33 | -0.09 | -0.32 | 0.14 |
| FE tighter | LUS | 0.855 | 0.893 | 1.27 | 1.06 | 1.53 | 1.10 | 0.92 | 1.32 | -0.09 | -0.31 | 0.15 |
| FE looser | LUS | 0.856 | 0.896 | 1.27 | 1.07 | 1.53 | 1.10 | 0.92 | 1.31 | -0.09 | -0.31 | 0.13 |
| LKJ4 | LUS | 0.855 | 0.896 | 1.27 | 1.06 | 1.52 | 1.10 | 0.92 | 1.31 | -0.08 | -0.31 | 0.13 |
| Baseline | REDS | 0.734 | 0.728 | 1.27 | 1.06 | 1.53 | 1.10 | 0.92 | 1.32 | -0.09 | -0.31 | 0.14 |
| SD tighter | REDS | 0.731 | 0.733 | 1.26 | 1.05 | 1.51 | 1.09 | 0.91 | 1.30 | -0.09 | -0.32 | 0.14 |
| SD looser | REDS | 0.733 | 0.726 | 1.28 | 1.07 | 1.54 | 1.11 | 0.93 | 1.33 | -0.09 | -0.32 | 0.14 |
| FE tighter | REDS | 0.715 | 0.723 | 1.27 | 1.06 | 1.53 | 1.10 | 0.92 | 1.32 | -0.09 | -0.31 | 0.15 |
| FE looser | REDS | 0.739 | 0.735 | 1.27 | 1.07 | 1.53 | 1.10 | 0.92 | 1.31 | -0.09 | -0.31 | 0.13 |
| LKJ4 | REDS | 0.740 | 0.733 | 1.27 | 1.06 | 1.52 | 1.10 | 0.92 | 1.31 | -0.08 | -0.31 | 0.13 |

The baseline model used weakly informative priors: fixed effects (FE) ~ Normal(0, 2.5); between-study standard deviations (SD) ~ Exponential(1); and the correlation between random effects ~ LKJ(2), where higher LKJ values imply stronger prior belief in independence.
Sensitivity analyses varied these priors as follows: SD tighter = Exponential(2); SD looser = Exponential(0.5); FE tighter = Normal(0, 1.5); FE looser = Normal(0, 3); and LKJ4 = LKJ(4).

# **Supplemental Methods**

## **Supplemental Methods 1.** Search strategies

Searches performed 9 April 2025

Total number of records identified	164983 records

Number of duplicates removed	8882 records

Number of records in final list	156101 records

Cochrane Central Register of Controlled Trials (2025, Issue 3 for CENTRAL and Issue 4 for Reviews, Protocols, and Clinical Answers) in the Cochrane Library (3758 hits)

#1	MeSH descriptor: [Ultrasonography] explode all trees

#2	(((lung* or pulmonar* or thora*) and (ultrason* or ultrasound* or "ultra sound" or echotomograph* or echo tomograph* or echograph* or sonograph* or acoustic)) or FLUS)

#3	MeSH descriptor: [Radiography, Thoracic] explode all trees

#4	((chest or lung* or thora*) near/3 (radiograph* or radiogram* or radiology or roentgen* or x‐ray* or (x NEXT ray*) or xray*))

#5	MeSH descriptor: [Tomography, X-Ray Computed] explode all trees

#6	((chest or thora*) NEAR/3 (CT or (computed NEXT tomograph*)))

#7	MeSH descriptor: [Remote Sensing Technology] explode all trees

#8	("Remote Dielectric Sensing" or ReDS)

#9	#1 or #2 or #3 or #4 or #5 or #6 or #7 or #8

#10	MeSH descriptor: [Heart Failure] explode all trees

#11	MeSH descriptor: [Oedema, Cardiac] explode all trees

#12	MeSH descriptor: [Pulmonary Oedema] explode all trees

#13	(((acute or severe or worsen* or decompensat* or destabili*) and (heart or cardiac) and (failure* or insufficienc*)) or ADHF or AHF)

#14	(((pulmonary or lung) and (oedema* or ooedema*)) or ((pulmonary or lung or systemic or heart) and congest*))

#15	MeSH descriptor: [Pleural Effusion] explode all trees

#16	(pleura* NEAR/5 (effusion* or fluid*))

#17	#10 or #11 or #12 or #13 or #14 or #15 or #16

#18	#9 and #17

MEDLINE Ovid (1946 to 9 April 2025) (38375 hits)

1.	exp Ultrasonography/

2.	(((lung* or pulmonar* or thora*) and (ultrason* or ultrasound* or "ultra sound" or echotomograph* or echo tomograph* or echograph* or sonograph* or acoustic)) or FLUS).ti,ab.

3.	exp Radiography, Thoracic/

4.	((chest or lung* or thora*) adj3 (radiograph* or radiogram* or radiology or roentgen* or "x‐ray*" or "x ray*" or xray*)).ti,ab.

5.	exp Tomography, X-Ray Computed/

6.	((chest or thora*) adj3 (CT or "computed tomograph*")).ti,ab.

7.	exp Remote Sensing Technology/

8.	("Remote Dielectric Sensing" or ReDS).ti,ab.

9.	1 or 2 or 3 or 4 or 5 or 6 or 7 or 8

10.	exp Heart Failure/

11.	exp Oedema, Cardiac/

12.	exp Pulmonary Oedema/

13.	(((acute or severe or worsen* or decompensat* or destabili*) and (heart or cardiac) and (failure* or insufficienc*)) or ADHF or AHF).ti,ab.

14.	(((pulmonary or lung) and (oedema* or ooedema*)) or ((pulmonary or lung or systemic or heart) and congest*)).ti,ab.

15.	exp Pleural Effusion/

16.	(pleura* adj5 (effusion* or fluid*)).ti,ab.

17.	10 or 11 or 12 or 13 or 14 or 15 or 16

18.	9 and 17

19.	18 not ((exp infant/ or exp child/) not (adolescent/ or exp adult/))

Embase Ovid (1974 to 9 April 2025) (109458 hits)

1.	exp echography/

2.	(((lung* or pulmonar* or thora*) and (ultrason* or ultrasound* or "ultra sound" or echotomograph* or echo tomograph* or echograph* or sonograph* or acoustic)) or FLUS).ti,ab.

3.	exp thorax radiography/

4.	((chest or lung* or thora*) adj3 (radiograph* or radiogram* or radiology or roentgen* or "x‐ray*" or "x ray*" or xray*)).ti,ab.

5.	exp x-ray computed tomography/

6.	((chest or thora*) adj3 (CT or "computed tomograph*")).ti,ab.

7.	exp remote sensing/

8.	("Remote Dielectric Sensing" or ReDS).ti,ab.

9.	1 or 2 or 3 or 4 or 5 or 6 or 7 or 8

10.	exp heart failure/

11.	exp heart oedema/

12.	exp lung oedema/

13.	(((acute or severe or worsen* or decompensat* or destabili*) and (heart or cardiac) and (failure* or insufficienc*)) or ADHF or AHF).ti,ab.

14.	(((pulmonary or lung) and (oedema* or ooedema*)) or ((pulmonary or lung or systemic or heart) and congest*)).ti,ab.

15.	exp pleura effusion/

16.	(pleura* adj5 (effusion* or fluid*)).ti,ab.

17.	10 or 11 or 12 or 13 or 14 or 15 or 16

18.	9 and 17

19.	18 not ((juvenile/ or exp child/) not (exp adult/ or exp adolescent/))

20.	limit 19 to "remove medline records"

LILACS (VHL Regional Portal; 1982 to 9 April 2025) (741 hits)

((mh:(ultrasonography OR e01.370.350.850 OR radiography, thoracic OR e01.370.350.700.730 OR tomography, x-ray computed OR e01.370.350.350.810 OR e01.370.350.600.350.700.810 OR e01.370.350.700.700.810 OR e01.370.350.700.810.810 OR e01.370.350.825.810.810 OR remote sensing technology OR e01.370.520.750.500 OR e05.925.500 OR l01.462.500.847.675.500 OR sp4.707.368.540.812)) OR ((((lung* OR pulmonar* OR thora*) AND (ultrason* OR ultrasound* OR "ultra sound" OR echotomograph* OR "echo tomograph*" OR echograph* OR sonograph* OR acoustic)) OR flus)) OR (((chest OR lung* OR thora*) AND (radiograph* OR radiogram* OR radiology OR roentgen* OR "x‐ray*" OR "x ray*" OR xray*))) OR (((chest OR thora*) AND (ct OR "computed tomograph*"))) OR (("Remote Dielectric Sensing" OR reds))) AND ((mh:(heart failure OR c14.280.434 OR oedema, cardiac OR c14.280.434.482 OR c23.888.277.197 OR pulmonary oedema OR c08.381.742 OR pleural effusion OR c08.528.652)) OR ((((acute OR severe OR worsen* OR decompensat* OR destabili*) AND (heart OR cardiac) AND (failure* OR insufficienc*)) OR adhf OR ahf)) OR ((((pulmonary OR lung) AND (oedema* OR ooedema*)) OR ((pulmonary OR lung OR systemic OR heart) AND congest*))) OR ((pleura* AND (effusion* OR fluid*)))) AND db:("LILACS")

Science Citation Index Expanded (1900 to 9 April 2025) and Conference Proceedings Citation Index – Science (1990 to 9 April 2025) (Web of Science) (12651 hits)

#10 #9 AND #5

#9 #6 OR #7 OR #8

#8 TS=(pleura* NEAR/5 (effusion* or fluid*))

#7 TS=(((pulmonary or lung) and (oedema* or ooedema*)) or ((pulmonary or lung or systemic or heart) and congest*))

#6 TS=(((acute or severe or worsen* or decompensat* or destabili*) and (heart or cardiac) and (failure* or insufficienc*)) or ADHF or AHF)

#5 #4 OR #3 OR #2 OR #1

#4 TS=("Remote Dielectric Sensing" or ReDS)

#3 TS=((chest or thora*) NEAR/3 (CT or (computed NEAR/0 tomograph*)))

#2 TS=((chest or lung* or thora*) near/3 (radiograph* or radiogram* or radiology or roentgen* or (x NEAR/1 ray*) or xray*))

#1 TS=(((lung* or pulmonar* or thora*) and (ultrason* or ultrasound* or "ultra sound" or echotomograph* or echo tomograph* or echograph* or sonograph* or acoustic)) or FLUS

## **Supplemental Methods 2.** Bayesian model specification

We fitted the bivariate model in R using the *brms* package, specifying two linked binomial components: one for true positives among disease-positive participants (sensitivity) and one for true negatives among disease-negative participants (specificity). Each component included the test modality and a study-level random effect. The random effects were specified using parallel notation in *brms*, which instructed the model to treat the study-level random effects for sensitivity and specificity as correlated. The two components were then combined into a bivariate.

We used weakly informative priors. These were specified as Normal(0, 2.5) distributions on the log-odds scale, which allowed a broad span of possible sensitivities and specificities. Between-study heterogeneity was modeled with Exponential(1) prior on the random-effect standard deviations, which assumes modest differences between studies while allowing larger variability when indicated by the data. The correlation between the study-level effects for sensitivity and specificity was assigned an LKJ(2) prior, which mildly down-weights extreme correlations while allowing either positive or negative dependence to be estimated from the data. Prior predictive checks indicated that our model gave equal probability to the full range of sensitivity and specificity values before any data are included. Supplementary Figure S13 show the predicted distribution of true positives (A) and true negatives (B) from 100 hypothetical positive and negative test results respectively.

Models were estimated using four Markov chains with 3,000 iterations each (1,500 warm-up). Convergence was confirmed by trace plots and R-hat values close to 1. Posterior means and 95%-credible intervals (CrI) were reported for each modality. Pairwise posterior differences were examined to determine whether the 95%CrI included 0. Sensitivity analyses were conducted with alternative prior specifications.

Below, we provide the R code used to implement the bivariate binomial model described above.

Model specification

bf_se <- bf(TP | trials(pos) ~ 0 + test + (1 | p | study), family = binomial())

bf_sp <- bf(TN | trials(neg) ~ 0 + test + (1 | p | study), family = binomial())

form_mv <- bf_se + bf_sp + set_rescor(FALSE)

Explanation

- TP | trials(pos) / TN | trials(neg):
  Binomial models for true positives among disease-positive participants and true negatives among disease-negative participants.
- 0 + test:
  Removes the intercept and estimates a separate fixed effect for each test modality.
- (1 | p | study):

Study-level random effects, specified with parallel notation (“p”), indicating that random effects are correlated across the two components.

- family = binomial():
  Specifies binomial likelihoods.
- bf_se + bf_sp:
  Combines the two components into a joint bivariate model.
- set_rescor(FALSE):
  Residual correlations not estimated for binomial outcomes.

Prior specification

priors <- c(

prior(normal(0, 2.5), class = "b", resp = "TP"),

prior(normal(0, 2.5), class = "b", resp = "TN"),

prior(exponential(1), class = "sd", resp = "TP"),

prior(exponential(1), class = "sd", resp = "TN"),

prior(lkj(2), class = "cor")

)

Explanation

- prior(normal(0, 2.5), class = "b", resp = "TP"/"TN")

Assigns Normal(0, 2.5) priors to the fixed-effect coefficients (class = "b") for the TP and TN.

- prior(exponential(1), class = "sd", resp = "TP"/"TN")

Specifies an Exponential(1) prior on the standard deviations (“sd”) of the study-level random effects.

- prior(lkj(2), class = "cor")
- LKJ(2) prior for the correlation (“cor”) between the random effects for TP and TN across studies.

Model fitting

fit <- brm(

form_mv,

data = df,

prior = priors,

chains = 4,

iter = 3000,

warmup = 1500,

seed = 42,

cores = max(1, parallel::detectCores() - 1),

control = list(adapt_delta = 0.95, max_treedepth = 12)

)

Explanation

- form_mv
  The joint bivariate model for sensitivity and specificity, as specified above.
- prior = priors
  Prior distributions for fixed effects, random effects, and correlations as defined in the prior specification.
- chains = 4
  Four independent Markov chains run in parallel to assess convergence.
- iter = 3000, warmup = 1500
  Each chain ran for 3,000 iterations, of which the first 1,500 were used for warm-up (adaptation) and discarded from the posterior summaries.
- seed = 42
  Sets the random number seed to ensure reproducibility.
- cores = max(1, parallel::detectCores() - 1)

Specifies the number of cores used for computation.

- control = list(adapt_delta = 0.95, max_treedepth = 12)
  Tuning parameters for the Hamiltonian Monte Carlo sampler.

# **Supplemental References**

**Reference list for all included studies**

1.	Aggarwal M, Gupta M, Vijan V, et al. Use of Lung Ultrasound For Diagnosing Acute Heart Failure in Emergency Department of Southern India. *J Clin Diagn Res*. Published online 2016. doi:10.7860/JCDR/2016/20661.8814

2.	Ali M, El Tahan S, El Nekidy AA, El Din Abdallah A, Salah K. Accuracy of point of care ultrasound in evaluation of patients with dyspnoea and chest pain in the emergency department. *Ann Clin Anal Med*. 2022;13(11). doi:10.4328/ACAM.21258

3.	Anderson KL, Jenq KY, Fields JM, Panebianco NL, Dean AJ. Diagnosing heart failure among acutely dyspneic patients with cardiac, inferior vena cava, and lung ultrasonography. *Am J Emerg Med*. 2013;31(8):1208-1214. doi:10.1016/j.ajem.2013.05.007

4.	Arthur M, Pichamuthu K, Turaka VP, et al. Bedside lung ultrasonography: comparison with chest radiography (BLUR), a diagnostic study in a developing country. *Postgrad Med J*. 2023;99(1173):724-730. doi:10.1136/pmj-2021-141343

5.	Azab NY, El Wahsh RA, Ammar ME, El-Koa AA. Role of chest ultrasonography in the diagnosis of the causes of acute respiratory failure. *Egypt J Chest Dis Tuberc*. 2022;71(3):301-305. doi:10.4103/ecdt.ecdt_51_21

6.	Baid H, Vempalli N, Kumar S, et al. Point of care ultrasound as initial diagnostic tool in acute dyspnoea patients in the emergency department of a tertiary care center: diagnostic accuracy study. *Int J Emerg Med*. 2022;15(1):27. doi:10.1186/s12245-022-00430-8

7.	Baker K, Mitchell G, Thompson AG, Stieler G. Comparison of a basic lung scanning protocol against formally reported chest x‐ray in the diagnosis of pulmonary ooedema. *Australas J Ultrasound Med*. 2013;16(4):183-189. doi:10.1002/j.2205-0140.2013.tb00245.x

8.	Baldi G, Gargani L, Abramo A, et al. Lung water assessment by lung ultrasonography in intensive care: a pilot study. *Intensive Care Med*. 2013;39(1):74-84. doi:10.1007/s00134-012-2694-x

9.	Balen F, Houze Cerfon CH, Lauque D, et al. Diagnostic performances of lung ultrasound associated with inferior vena cava assessment for the diagnosis of acute heart failure in elderly emergency patients: a diagnostic study. *Eur J Emerg Med*. 2021;28(2):119-124. doi:10.1097/MEJ.0000000000000759

10.	Bataille B, Riu B, Ferre F, et al. Integrated Use of Bedside Lung Ultrasound and Echocardiography in Acute Respiratory Failure. *Chest*. 2014;146(6):1586-1593. doi:10.1378/chest.14-0681

11.	Bekgoz B, Kilicaslan I, Bildik F, et al. BLUE protocol ultrasonography in Emergency Department patients presenting with acute dyspnoea. *Am J Emerg Med*. 2019;37(11):2020-2027. doi:10.1016/j.ajem.2019.02.028

12.	Bitar Z, Maadarani O, Almerri K. Sonographic chest B-lines anticipate elevated B-type natriuretic peptide level, irrespective of ejection fraction. *Ann Intensive Care*. 2015;5(1):56. doi:10.1186/s13613-015-0100-x

13.	Bouzidi H, Hammami S, Zairi I, et al. Role of pulmonary ultrasonography in diagnosis of acute heart failure. *Curr Probl Cardiol*. 2025;50(1):102910. doi:10.1016/j.cpcardiol.2024.102910

14.	Buessler A, Chouihed T, Duarte K, et al. Accuracy of Several Lung Ultrasound Methods for the Diagnosis of Acute Heart Failure in the ED. *Chest*. 2020;157(1):99-110. doi:10.1016/j.chest.2019.07.017

15.	Buhumaid RE, St-Cyr Bourque J, Shokoohi H, Ma IWY, Longacre M, Liteplo AS. Integrating point-of-care ultrasound in the ED evaluation of patients presenting with chest pain and shortness of breath. *Am J Emerg Med*. 2019;37(2):298-303. doi:10.1016/j.ajem.2018.10.059

16.	Carlino MV, Paladino F, Sforza A, et al. Assessment of left atrial size in addition to focused cardiopulmonary ultrasound improves diagnostic accuracy of acute heart failure in the Emergency Department. *Echocardiography*. 2018;35(6):785-791. doi:10.1111/echo.13851

17.	Chaitra S, Hattiholi VV. Diagnostic Accuracy of Bedside Lung Ultrasound in Emergency Protocol for the Diagnosis of Acute Respiratory Failure. *J Med Ultrasound*. 2022;30(2):94-100. doi:10.4103/JMU.JMU_25_21

18.	Chiem AT, Chan CH, Ander DS, Kobylivker AN, Manson WC. Comparison of Expert and Novice Sonographers’ Performance in Focused Lung Ultrasonography in Dyspnoea (FLUID) to Diagnose Patients With Acute Heart Failure Syndrome. Costantino T, ed. *Acad Emerg Med*. 2015;22(5):564-573. doi:10.1111/acem.12651

19.	Cibinel GA, Casoli G, Elia F, et al. Diagnostic accuracy and reproducibility of pleural and lung ultrasound in discriminating cardiogenic causes of acute dyspnoea in the Emergency Department. *Intern Emerg Med*. 2012;7(1):65-70. doi:10.1007/s11739-011-0709-1

20.	Coiro S, Porot G, Rossignol P, et al. Prognostic value of pulmonary congestion assessed by lung ultrasound imaging during heart failure hospitalisation: A two-centre cohort study. *Sci Rep*. 2016;6(1):39426. doi:10.1038/srep39426

21.	De Carvalho H, Javaudin F, Le Bastard Q, Boureau AS, Montassier E, Le Conte P. Effect of chest ultrasound on diagnostic workup in elderly patients with acute respiratory failure in the emergency department: a prospective study. *Eur J Emerg Med*. 2021;28(1):29-33. doi:10.1097/MEJ.0000000000000732

22.	Dehbozorgi A, Eslami Nejad S, Mousavi-roknabadi RS, Sharifi M, Tafakori A, Jalli R. Lung and cardiac ultrasound (LuCUS) protocol in diagnosing acute heart failure in patients with acute dyspnoea. *Am J Emerg Med*. 2019;37(11):2055-2060. doi:10.1016/j.ajem.2019.02.040

23.	Dexheimer Neto FL, Andrade JMSD, Raupp ACT, et al. Diagnostic accuracy of the Bedside Lung Ultrasound in Emergency protocol for the diagnosis of acute respiratory failure in spontaneously breathing patients,. *J Bras Pneumol*. 2015;41(1):58-64. doi:10.1590/S1806-37132015000100008

24.	Elatroush HH, Essawy TS, Kenawy MM, Karoub ASAEA, Ismail AM. The Assessment of the Diagnostic Accuracy of Bedside Lung Ultrasound in Critically Ill Respiratory Failure Patients. *Biomed Pharmacol J*. 2023;16(1):525-532. doi:10.13005/bpj/2634

25.	Elmahalawy II, Doha NM, Ebeid OM, Abdel-Hady MA, Saied O. Role of thoracic ultrasound in diagnosis of pulmonary and pleural diseases in critically ill patients. *Egypt J Chest Dis Tuberc*. 2017;66(2):261-266. doi:10.1016/j.ejcdt.2016.10.005

26.	Elnaem WH, Tammam HM, Zidan MA, Mahmoud MI. The relative efficacy of chest ultrasonography in comparison to other diagnostic modalities in the evaluation of dyspneic patient. *Egypt J Chest Dis Tuberc*. 2017;66(1):165-168. doi:10.1016/j.ejcdt.2016.12.005

27.	Ely W, Smith AC, Chiles C, et al. Radiologic determination of intravascular volume status using portable, digital chest radiography: A prospective investigation in 100 patients: *Crit Care Med*. 2001;29(8):1502-1512. doi:10.1097/00003246-200108000-00002

28.	Farahmand S, Abdolhoseini A, Aliniagerdroudbari E, Babaniamansour S, Baratloo A, Bagheri-Hariri S. Point-of-care ultrasound modalities in terms of diagnosing acute decompensated heart failure in emergency department; a diagnostic accuracy study. *Intern Emerg Med*. 2020;15(3):491-499. doi:10.1007/s11739-019-02233-x

29.	Feuerstein D, Schmid B, Mieves M, Fink K, Busche C, Busch HJ. Reduktion von Röntgenuntersuchungen der Lunge bei Patienten mit Atemnot durch ein ultraschallgestütztes Protokoll. *Intensiv Notfallbehandlung*. 2022;47(07):130-133. doi:10.5414/IBX0579

30.	Gallard E, Redonnet JP, Bourcier JE, et al. Diagnostic performance of cardiopulmonary ultrasound performed by the emergency physician in the management of acute dyspnoea. *Am J Emerg Med*. 2015;33(3):352-358. doi:10.1016/j.ajem.2014.12.003

31.	Gargani L, Frassi F, Soldati G, Tesorio P, Gheorghiade M, Picano E. Ultrasound lung comets for the differential diagnosis of acute cardiogenic dyspnoea: A comparison with natriuretic peptides☆. *Eur J Heart Fail*. 2008;10(1):70-77. doi:10.1016/j.ejheart.2007.10.009

32.	Ghauri, Khawaja Junaid Mustafa, Arslaan Javaeed, Abdus Salam Khan. Accuracy of lung ultrasound and chest X-rays in diagnosing acute pulmonary ooedema in patients presenting with acute dyspnoea in emergency department. *J Pak Med Assoc*. 2021;71(10):2423-2425. doi:10.47391/JPMA.03-453

33.	Gillespie ND, McNeill G, Pringle T, Ogston S, Struthers AD, Pringle SD. Cross sectional study of contribution of clinical assessment and simple cardiac investigations to diagnosis of left ventricular systolic dysfunction in patients admitted with acute dyspnoea. *BMJ*. 1997;314(7085):936-936. doi:10.1136/bmj.314.7085.936

34.	Glöckner E, Wening F, Christ M, Dechêne A, Singler K. Lung Ultrasound Eight-Point Method in Diagnosing Acute Heart Failure in Emergency Patients with Acute Dyspnoea: Diagnostic Accuracy and 72 h Monitoring. *Medicina (Mex)*. 2020;56(8):379. doi:10.3390/medicina56080379

35.	Heldeweg MLA, Smit MR, Kramer-Elliott SR, et al. Lung Ultrasound Signs to Diagnose and Discriminate Interstitial Syndromes in ICU Patients: A Diagnostic Accuracy Study in Two Cohorts*. *Crit Care Med*. 2022;50(11):1607-1617. doi:10.1097/CCM.0000000000005620

36.	Hubert A, Girerd N, Le Breton H, et al. Diagnostic accuracy of lung ultrasound for identification of elevated left ventricular filling pressure. *Int J Cardiol*. 2019;281:62-68. doi:10.1016/j.ijcard.2019.01.055

37.	Imamura T, Hori M, Ueno Y, et al. Association between Lung Fluid Levels Estimated by Remote Dielectric Sensing Values and Invasive Hemodynamic Measurements. *J Clin Med*. 2022;11(5):1208. doi:10.3390/jcm11051208

38.	Inui G, Tomita K, Fukuki M, et al. Clinical characteristics for distinguishing between acute cardiogenic pulmonary oedema and community-acquired pneumonia in elderly patients: a prospective observational study. *Monaldi Arch Chest Dis*. Published online August 1, 2023. doi:10.4081/monaldi.2023.2633

39.	Izumida T, Imamura T, Kinugawa K. Remote dielectric sensing and lung ultrasound to assess pulmonary congestion. *Heart Vessels*. 2023;38(4):517-522. doi:10.1007/s00380-022-02190-0

40.	Izumida T, Imamura T, Hori M, Kinugawa K. Correlation Between Plasma B-Type Natriuretic Peptide Levels and Remote Dielectric Sensing in Patients with Heart Failure. *Int Heart J*. 2022;63(6):1128-1133. doi:10.1536/ihj.22-348

41.	Johannessen Ø, Uthaug Reite F, Bhatnagar R, Øvrebotten T, Einvik G, Myhre PL. Lung Ultrasound to Assess Pulmonary Congestion in Patients with Acute Exacerbation of COPD. *Int J Chron Obstruct Pulmon Dis*. 2023;Volume 18:693-703. doi:10.2147/COPD.S396855

42.	Joseph RB, Kumar AKAS, James A. A Comparative Study on Lung Ultrasound Compared to Chest X-Ray in Diagnosis of Patients Admitted to Critical Care Department with Acute Respiratory Failure. *Eur J Cardiovasc Med*. 2024;14(1):80-87.

43.	Kennedy S, Simon B, Alter HJ, Cheung P. Ability of Physicians to Diagnose Congestive Heart Failure Based on Chest X-Ray. *J Emerg Med*. 2011;40(1):47-52. doi:10.1016/j.jemermed.2009.10.018

44.	Knudsen CW, Omland T, Clopton P, et al. Diagnostic value of B-Type natriuretic peptide and chest radiographic findings in patients with acute dyspnoea. *Am J Med*. 2004;116(6):363-368. doi:10.1016/j.amjmed.2003.10.028

45.	Koh Y, Chua MT, Ho WH, Lee C, Chan GWH, Sen Kuan W. Assessment of dyspneic patients in the emergency department using point-of-care lung and cardiac ultrasonography—a prospective observational study. *J Thorac Dis*. 2018;10(11):6221-6229. doi:10.21037/jtd.2018.10.30

46.	Lajili F, Toumia M, Sekma A, et al. Value of Lung Ultrasound Sonography B-Lines Quantification as a Marker of Heart Failure in COPD Exacerbation. *Int J Chron Obstruct Pulmon Dis*. 2024;Volume 19:1767-1774. doi:10.2147/COPD.S447819

47.	Laursen CB, Hänselmann A, Posth S, Mikkelsen S, Videbæk L, Berg H. Prehospital lung ultrasound for the diagnosis of cardiogenic pulmonary ooedema: a pilot study. *Scand J Trauma Resusc Emerg Med*. 2016;24(1):96. doi:10.1186/s13049-016-0288-2

48.	Lichtenstein DA, Mezière GA. Relevance of Lung Ultrasound in the Diagnosis of Acute Respiratory Failure*: The BLUE Protocol. *Chest*. 2008;134(1):117-125. doi:10.1378/chest.07-2800

49.	Lichtenstein DA, Mezière GA, Lagoueyte JF, Biderman P, Goldstein I, Gepner A. A-Lines and B-Lines. *Chest*. 2009;136(4):1014-1020. doi:10.1378/chest.09-0001

50.	Liteplo AS, Marill KA, Villen T, et al. Emergency Thoracic Ultrasound in the Differentiation of the Etiology of Shortness of Breath (ETUDES): Sonographic B-lines and N-terminal Pro-brain-type Natriuretic Peptide in Diagnosing Congestive Heart Failure. *Acad Emerg Med*. 2009;16(3):201-210. doi:10.1111/j.1553-2712.2008.00347.x

51.	Liu Z peng, Zhang Y, Bian H, et al. Clinical application of rapid B-line score with lung ultrasonography in differentiating between pulmonary infection and pulmonary infection with acute left ventricular heart failure. *Am J Emerg Med*. 2016;34(2):278-281. doi:10.1016/j.ajem.2015.10.050

52.	Martinez-Rumayor AA, Vazquez J, Rehman SU, Januzzi JL. Relative value of amino-terminal pro-B-type natriuretic peptide testing and radiographic standards for the diagnostic evaluation of heart failure in acutely dyspneic subjects. *Biomarkers*. 2010;15(2):175-182. doi:10.3109/13547500903411087

53.	Mayr U, Lukas M, Habenicht L, et al. B-Lines Scores Derived From Lung Ultrasound Provide Accurate Prediction of Extravascular Lung Water Index: An Observational Study in Critically Ill Patients. *J Intensive Care Med*. 2022;37(1):21-31. doi:10.1177/0885066620967655

54.	Miger KC, Fabricius-Bjerre A, Maschmann CP, et al. Clinical Applicability of Lung Ultrasound Methods in the Emergency Department to Detect Pulmonary Congestion on Computed Tomography. *Ultraschall Med*. 2021;42(3):21-30. doi:10.1055/A-1021-1470/ID/JR022-27

55.	Miger K, Overgaard Olesen AS, Grand J, et al. Computed tomography or chest X-ray to assess pulmonary congestion in dyspnoeic patients with acute heart failure. *ESC Heart Fail*. 2024;11(2):1163-1173. doi:10.1002/ehf2.14688

56.	Modi U, Pa D, Rr D, Gs P, Hr D. Utility of Bedside Lung Ultrasound in Emergency (Blue) Protocol as a Diagnostic Tool in Patient with Acute Respiratory Distress Presenting in Emergency Department. *Int J Pharm Clin Res*.

57.	Msolli MA, Sekma A, Marzouk MB, et al. Bedside lung ultrasonography by emergency department residents as an aid for identifying heart failure in patients with acute dyspnoea after a 2-h training course. *Ultrasound J*. 2021;13(1):5. doi:10.1186/s13089-021-00207-9

58.	Mueller-Lenke N, Rudez J, Staub D, et al. Use of chest radiography in the emergency diagnosis of acute congestive heart failure: Table 1. *Heart*. 2006;92(5):695-696. doi:10.1136/hrt.2005.074583

59.	Mumoli N, Vitale J, Giorgi-Pierfranceschi M, et al. Accuracy of Nurse-Performed Lung Ultrasound in Patients With Acute Dyspnoea: A Prospective Observational Study. *Medicine (Baltimore)*. 2016;95(9):e2925. doi:10.1097/MD.0000000000002925

60.	Nagaharu K, Tsumura N, Itoh T, Murata T. Diagnostic utility of lung echography for congestive heart failure performed by junior resident doctors. *J Gen Fam Med*. 2022;23(6):401-406. doi:10.1002/jgf2.575

61.	Nakao S, Vaillancourt C, Taljaard M, Nemnom MJ, Woo MY, Stiell IG. Diagnostic Accuracy of Lung Point-Of-Care Ultrasonography for Acute Heart Failure Compared With Chest X-Ray Study Among Dyspneic Older Patients in the Emergency Department. *J Emerg Med*. 2021;61(2):161-168. doi:10.1016/j.jemermed.2021.02.019

62.	Neeraj G, Gopal V, Vadlani V, Diwakarla V, Arif. Efficacy of BLUE Protocol in Diagnosing Respiratory Failure in Patients Attending a Multidisciplinary ICU In a Teaching Hospital. *Eur J Cardiovasc Med*. 13(4).

63.	Ocel S, Kekec Z, Taskin O, Belli F, Tugcan MO. Diagnostic role of thoracic ultrasound in patients with acute respiratory failure at emergency service. *Ir J Med Sci 1971 -*. 2024;193(3):1573-1579. doi:10.1007/s11845-024-03618-4

64.	Öhman J, Harjola VP, Karjalainen P, Lassus J. Rapid cardiothoracic ultrasound protocol for diagnosis of acute heart failure in the emergency department. *Eur J Emerg Med*. 2019;26(2):112-117. doi:10.1097/MEJ.0000000000000499

65.	Olesen ASO, Miger K, Fabricius-Bjerre A, et al. Remote dielectric sensing to detect acute heart failure in patients with dyspnoea: a prospective observational study in the emergency department. Bäck M, ed. *Eur Heart J Open*. 2022;2(6):oeac073. doi:10.1093/ehjopen/oeac073

66.	Panda R, Saigal S, Joshi R, et al. Accuracy of Critical Care Ultrasonography Plus Arterial Blood Gas Analysis Based Algorithm in Diagnosing Aetiology of Acute Respiratory Failure. *J Crit Care Med*. 2023;9(1):20-29. doi:10.2478/jccm-2023-0006

67.	Patel C, Bhatt H, Parikh S, Jhaveri B, Puranik J. Bedside lung ultrasound in emergency protocol as a diagnostic tool in patients of acute respiratory distress presenting to emergency department. *J Emerg Trauma Shock*. 2018;11(2):125. doi:10.4103/JETS.JETS_21_17

68.	Perrone T, Maggi A, Sgarlata C, et al. Lung ultrasound in internal medicine: A bedside help to increase accuracy in the diagnosis of dyspnoea. *Eur J Intern Med*. 2017;46:61-65. doi:10.1016/j.ejim.2017.07.034

69.	Pivetta E, Goffi A, Lupia E, et al. Lung Ultrasound-Implemented Diagnosis of Acute Decompensated Heart Failure in the ED. *Chest*. 2015;148(1):202-210. doi:10.1378/chest.14-2608

70.	Prosen G, Klemen P, Strnad M, Grmec Š. Combination of lung ultrasound (a comet-tail sign) and N-terminal pro-brain natriuretic peptide in differentiating acute heart failure from chronic obstructive pulmonary disease and asthma as cause of acute dyspnoea in prehospital emergency setting. *Crit Care*. 2011;15(2):R114. doi:10.1186/cc10140

71.	Rafique Z, McArthur R, Sekhon N, Mesbah H, Almasary A, Peacock WF. Remote dielectric sensing for detecting pulmonary oedema in the emergency department. *Am J Emerg Med*. 2022;55:11-15. doi:10.1016/j.ajem.2022.02.030

72.	Russell FM, Supples M, Tamhankar O, Liao M, Finnegan P. Prehospital lung ultrasound in acute heart failure: Impact on diagnosis and treatment. *Acad Emerg Med*. 2024;31(1):42-48. doi:10.1111/acem.14811

73.	Russell FM, Ehrman RR. A Modified Lung and Cardiac Ultrasound Protocol Saves Time and Rules in the Diagnosis of Acute Heart Failure. *J Emerg Med*. 2017;52(6):839-845. doi:10.1016/j.jemermed.2017.02.003

74.	Saboo R, Chand D. A Study of NT-ProBNP and ETCO_2_ in Patients Presenting with Acute Dyspnoea. *Vidarbha J Intern Med*. 2022;32:94-99. doi:10.25259/VJIM_19_2022

75.	Sartini S, Frizzi J, Borselli M, et al. Which method is best for an early accurate diagnosis of acute heart failure? Comparison between lung ultrasound, chest X-ray and NT pro-BNP performance: a prospective study. *Intern Emerg Med*. 2017;12(6):861-869. doi:10.1007/s11739-016-1498-3

76.	Schoeneck J, Coughlin R, Baloescu C, et al. Paramedic-performed Prehospital Point-of-care Ultrasound for Patients with Undifferentiated Dyspnoea: A Pilot Study. *West J Emerg Med*. 2021;22(3). doi:10.5811/westjem.2020.12.49254

77.	Secko M, Cheng Y, Raj S, et al. Comprehensive Physical Exam versus Lung Ultrasound for Dyspneic Patients in the Emergency Department. Preprint posted online October 10, 2022. doi:10.1101/2022.10.08.22280828

78.	Seibel A, Zechner PM, Berghold A, et al. B‐Lines for the assessment of extravascular lung water: Just focused or semi‐quantitative? *Acta Anaesthesiol Scand*. 2020;64(7):953-960. doi:10.1111/aas.13586

79.	Sekiguchi H, Schenck LA, Horie R, et al. Critical Care Ultrasonography Differentiates ARDS, Pulmonary Oedema, and Other Causes in the Early Course of Acute Hypoxemic Respiratory Failure. *Chest*. 2015;148(4):912-918. doi:10.1378/chest.15-0341

80.	Sen S, Acash G, Sarwar A, Lei Y, Dargin JM. Utility and diagnostic accuracy of bedside lung ultrasonography during medical emergency team (MET) activations for respiratory deterioration. *J Crit Care*. 2017;40:58-62. doi:10.1016/j.jcrc.2017.03.007

81.	Sforza A, Mancusi C, Carlino MV, et al. Diagnostic performance of multi-organ ultrasound with pocket-sized device in the management of acute dyspnoea. *Cardiovasc Ultrasound*. 2017;15(1):16. doi:10.1186/s12947-017-0105-8

82.	Sharma A, Kaeley N, Aravindan N, Datta SS, Vasisht S. Using Lung Ultrasound Combined With N-terminal Pro-brain Natriuretic Peptide to Differentiate Acute Heart Failure From Chronic Obstructive Pulmonary Disease and Asthma in Emergency Department Patients Experiencing Acute Shortness of Breath. *Cureus*. Published online January 9, 2025. doi:10.7759/cureus.77171

83.	Siddiqi A. DIAGNOSTIC ACCURACY OF POINT OF CARE ULTRASOUND IN ADULT PATIENTS PRESENTING WITH ACUTE DYSPNOEA TO EMERGENCY DEPARTMENT. *J Popul Ther Clin*. Published online February 15, 2025:939-944. doi:10.53555/ytxrdn57

84.	Studler U, Kretzschmar M, Christ M, et al. Accuracy of chest radiographs in the emergency diagnosis of heart failure. *Eur Radiol*. 2008;18(8):1644-1652. doi:10.1007/s00330-008-0930-0

85.	Tseverava M, Tseverava D. “Comet Tail” Artefact in Diagnosis of Pulmonary Congestion in Patients with Diastolic Heart Failure. *Georgian Med News*. 2010;181(10):28-35.

86.	Tækker M, Kristjánsdóttir B, Andersen MB, et al. Diagnostic accuracy of ultra-low-dose chest computed tomography in an emergency department. *Acta Radiol*. 2022;63(3):336-344. doi:10.1177/0284185121995804

87.	Uriel N, Sayer G, Imamura T, et al. Relationship between noninvasive assessment of lung fluid volume and invasively measured cardiac hemodynamics. *J Am Heart Assoc*. 2018;7(22). doi:10.1161/JAHA.118.009175

88.	Vauthier C, Chabannon M, Markarian T, et al. Rendimiento de un algoritmo basado en ecografía cardiopulmonar a la cabecera del paciente (POCUS) para el diagnóstico de insuficiencia cardiaca aguda en pacientes que consultan en urgencias por disnea aguda.

89.	Vitturi N, Soattin M, Allemand E, Simoni F, Realdi G. Thoracic ultrasonography: A new method for the work-up of patients with dyspnoea. *J Ultrasound*. 2011;14(3):147-151. doi:10.1016/j.jus.2011.06.009

90.	Volpicelli G, Carpinteri G, Anile A. Lung Ultrasound Predicts Well Extravascular Lung Water but Is of Limited Usefulness in the Prediction of Wedge Pressure. *Crit CARE Med*.

91.	Wooten WM, Shaffer LET, Hamilton LA. Bedside Ultrasound Versus Chest Radiography for Detection of Pulmonary Oedema: A Prospective Cohort Study. *J Ultrasound Med*. 2019;38(4):967-973. doi:10.1002/jum.14781

92.	Yang F, Wang Q, Zhi G, et al. The application of lung ultrasound in acute decompensated heart failure in heart failure with preserved and reduced ejection fraction. *Echocardiography*. 2017;34(10):1462-1469. doi:10.1111/echo.13646

93.	Zhong L, Su D chun, Wang K, Zhang S long, Cong T. Transthoracic lung ultrasound displaying comet-tail sign in diagnosis of cardiogenic dyspnoea. *Chin J Med Imaging Technol*. 2013;29(4):561-564. doi:10.13929/j.1003-3289.2013.04.011

94.	Zhou S, Zha Y, Wang C, Wu J, Liu W, Liu B. [The clinical value of bedside lung ultrasound in the diagnosis of chronic obstructive pulmonary disease and cardiac pulmonary oedema]. *Zhonghua Wei Zhong Bing Ji Jiu Yi Xue*. 2014;26(8):558-562. doi:10.3760/cma.j.issn.2095-4352.2014.08.007

95.	Zhu L, Zhong H, Liu P, Xu Z. Point-of-care lung ultrasound optimization scheme for the assessment of cardiogenic pulmonary oedema: A promising tool. *J Radiat Res Appl Sci*. 2024;17(2):100859. doi:10.1016/j.jrras.2024.100859
